# Light‐Triggered Disassembly of Peptide Nanostructures

**DOI:** 10.1002/cbic.202500414

**Published:** 2025-08-14

**Authors:** Raphael Meyer, Julian Link, Lucas Gunkel, Albin Lahu, Hakan Demirezen, Tanja Weil, David Y. W. Ng

**Affiliations:** ^1^ Department of the Synthesis of Macromolecule Max Planck Institute for Polymer Research Ackermannweg 10 55128 Mainz Germany; ^2^ Department of Molecular Spectroscopy Max Planck Institute for Polymer Research Ackermannweg 10 55128 Mainz Germany

**Keywords:** disassembly, peptide amphiphile, photochemistry, photodimerization, supramolecular assembly

## Abstract

While the assembly of supramolecular peptide nanostructures with diverse functions and morphologies has been extensively studied, the controlled disassembly of these architectures remains less understood. To address this, two short amphiphilic peptides incorporating anthracene as a light‐responsive unit and lysine as a pH‐sensitive residue are designed. These peptides self‐assemble into nanosheets or nanoribbons with distinct secondary structures, which are further tunable by pH through modulation of peptide charge. Upon irradiation at 365 nm, the anthracene moieties undergo a bimolecular [4 + 4] cycloaddition, disrupting the π–π stacking interactions by distorting the planarity of the aromatic units. This photoreaction leads to disassembly of the supramolecular architectures within 10 min. Unlike monomolecular reactions such as photocleavage, the kinetics of this bimolecular photodimerization are significantly influenced by the degree of preorganization within the assemblies. These findings underscore the critical interplay between supramolecular architecture and molecular photochemistry, enabling light‐triggered, structure‐dependent disassembly of diverse peptide nanostructures.

## Graphical abstract

Two amphiphilic peptides incorporating anthracene as a light‐responsive moiety that assemble into 2D‐sheets or ribbons are designed. These nanostructures are disassembled upon irradiation with 365 nm light by bimolecular [4 + 4] cycloaddition. The reaction kinetics are depended on peptide preorganization, linking structure to reactivity.

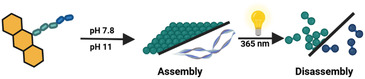

## 1 Introduction

Supramolecular peptide nanostructures have emerged as versatile biomaterials,^[^ ^1^ ^,^ ^2^ ^]^ addressing a broad range of applications in biomedicine^[^ ^3^ ^]^ and tissue engineering^[^ ^4^ ^]^ as they offer scaffolds that can be customized with diverse functionalities. Moreover, their synthesis routes are well‐developed with the advent of modern biorthogonal chemistries, thus bringing forth the next generation of materials that exhibit various depths of adaptivity, mechanochemical, and viscoelastic properties.^[^ ^5^, ^6^ ^–^ ^7^ ^]^ Despite these seminal advancements in the supramolecular assembly of peptide materials,^[^ ^8^, ^9^, ^10^, ^11^, ^12^ ^–^ ^13^ ^]^ their disassembly upon completion of their designated tasks, while equally critical, is much less explored.

One of the main reasons is that a large proportion of supramolecular peptide materials operates dominantly on *β*‐sheet type interactions found in neurodegenerative amyloid plaques and pathogenic protein aggregates.^[^ ^14^, ^15^ ^–^ ^16^ ^]^ The combined synergy between hydrogen bonding, *π*–*π* stacking, and hydrophobic forces results in the aggregate or material exhibiting high thermodynamic stability. Hence, disassembly strategies would need to overcome a tremendous energy barrier to break apart the strong attractive forces holding them together. Using the concept of inducing molecular, geometric frustration to the aggregate by multiple strong local interactions, the development of small molecular *β*‐sheet breakers^[^ ^17^ ^]^ or molecular tweezers^[^ ^18^ ^,^ ^19^ ^]^ has found preliminary successes. However, while these disassembling molecules are attractive as therapeutic solutions, they are cumbersome for material‐based applications where a more integrative design is required for processing.

In this regard, supramolecular peptide scaffolds containing stimulus–responsive groups (pH,^[^ ^20^ ^]^ temperature,^[^ ^21^ ^]^ enzymes,^[^ ^22^ ^]^ redox reactions,^[^ ^23^ ^]^ or light^[^ ^24^ ^,^ ^25^ ^]^) that chemically react, cleave, or isomerize have been exploited to disrupt intermolecular forces on demand. Among these, light offers a unique advantage in self‐assembly processes as photons do not directly interfere with intermolecular forces, and the resultant photochemical reactions can be precisely controlled spatiotemporally.^[^ ^26^ ^]^ Therefore, light‐responsive systems offer promising opportunities for biological applications, such as controlled drug release^[^ ^27^ ^]^ or harnessing light energy to generate heat or reactive oxygen species for use in photothermal or photodynamic therapy.^[^ ^28^, ^29^ ^–^ ^30^ ^]^ In this regard, photoswitches (i.e., azobenzenes, spiropyranes) and photocleavable groups have been developed to control the aggregation threshold of a targeted self‐assembling peptide.^[^ ^26^ ^]^ The resultant light‐induced chemical change occurs within the self‐assembling molecule (monomolecular), where structural isomerization or cleavage destabilizes the formed aggregate, favoring its dissolution. Using these motifs allow reactions to occur homogeneously, with every molecule exposed to light behaving in a similar fashion.^[^ ^31^ ^]^

In contrast, bimolecular photocycloaddition reactions, such as the [4 + 4] photodimerization of anthracene, are dependent on how molecules are preorganized within the solid‐state aggregate. Anthracene molecules that are stacked and sufficiently oriented to facilitate the cycloaddition will afford successful dimerization, leading to a distortion of the planar anthracene groups and disrupting the constructive *π*‐*π* interactions. The geometric frustration generated as a consequence has recently been shown to induce morphological switching in the nanoscale, between fibers and vesicles, or the bending of nanotubes.^[^ ^32^ ^,^ ^33^ ^]^ Nonetheless, the extent of how 1D and 2D nanoscale structures succumb to the molecular deformation to allow structure‐selective disassembly has not been explored.

**Figure 1:**
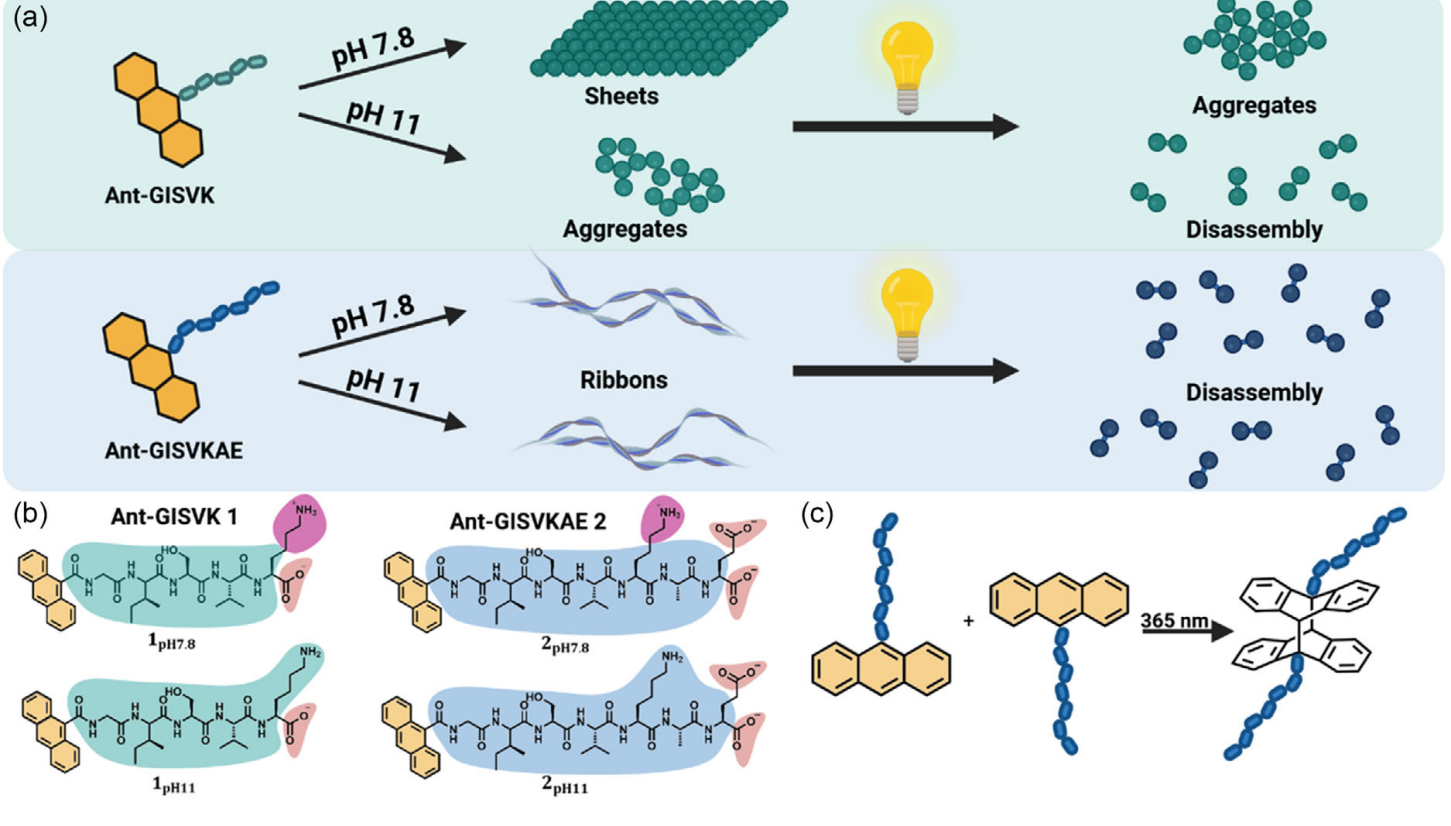
a) Schematic illustration of the assembly pathways of 1 and 2 at different pH and the disassembly pathway upon irradiation. b) Chemical structure of 1 and 2, respectively, at pH 7.8 or pH 11, showing the different charged states. c) [4 + 4] photodimerization of anthracene showing the bent configuration of the dimer.

In this study, we designed two anthracene‐containing, short amphiphilic peptides, Anthracene‐GISVK (Ant‐GISVK) and Ant‐GISVKAE, to assemble different pH‐dependent *β*‐sheet nanostructures, imparted by the lysine (K) and glutamic acid (E) residues (**Figure** **1a**). At pH 7.8 and pH 11, the overall charge on the peptides can be modulated, which in turn dictates molecular preorganization within the assembly. Subsequently, the kinetics of the photodimerization of anthracene (Figure 1c) are monitored via UV–vis spectroscopy to correlate the reaction efficiency and the extent of structural disruption. The loss of secondary structure integrity and eventual disassembly are investigated via circular dichroism (CD) spectroscopy and transmission electron microscopy (TEM).

## 2 Results and Discussion

Ant‐GISVK **1** and Ant‐GISVKAE **2** (Figure 1b) were synthesized using Fmoc‐solid phase peptide synthesis on Wang resin using *N,N*′‐diisopropylcarbodiimide (DIC) and ethyl (2*Z*)‐2‐cyano‐2‐(hydroxyimino)acetate (OxymaPure) for the iterative peptide coupling steps and benzotriazol‐1‐yloxytripyrrolidino‐phosphonium hexafluoro‐phosphate (PyBOP) and diisopropylethylamine (DIPEA) for the coupling of 9‐anthracenecarboxylic acid to the peptide, respectively. Purification was achieved by HPLC, and the peptides subsequently were analyzed by nuclear magnetic resonance (NMR) spectroscopy and liquid chromatography‐mass spectrometry (LCMS) (Figure S1–6, Supporting Information).

The peptides were then assembled in a solvent mixture of 5% DMSO in 95% NH_4_HCO_3_ buffer (10 mM) at pH 7.8 and pH 11 (adjusted with 1 M NaOH), respectively. Raising the pH to 11 results in the deprotonation of the side chain lysine (pKa = 10.4),^[^ ^34^ ^]^ thereby, causing the overall charge of both peptides to become more negative compared to pH 7.8, where the lysine is positively charged (Figure 1b). Subsequently, we performed TEM of the peptides at both pH values and analyzed the influence of charge on the assembled nanostructure.

**Figure 2:**
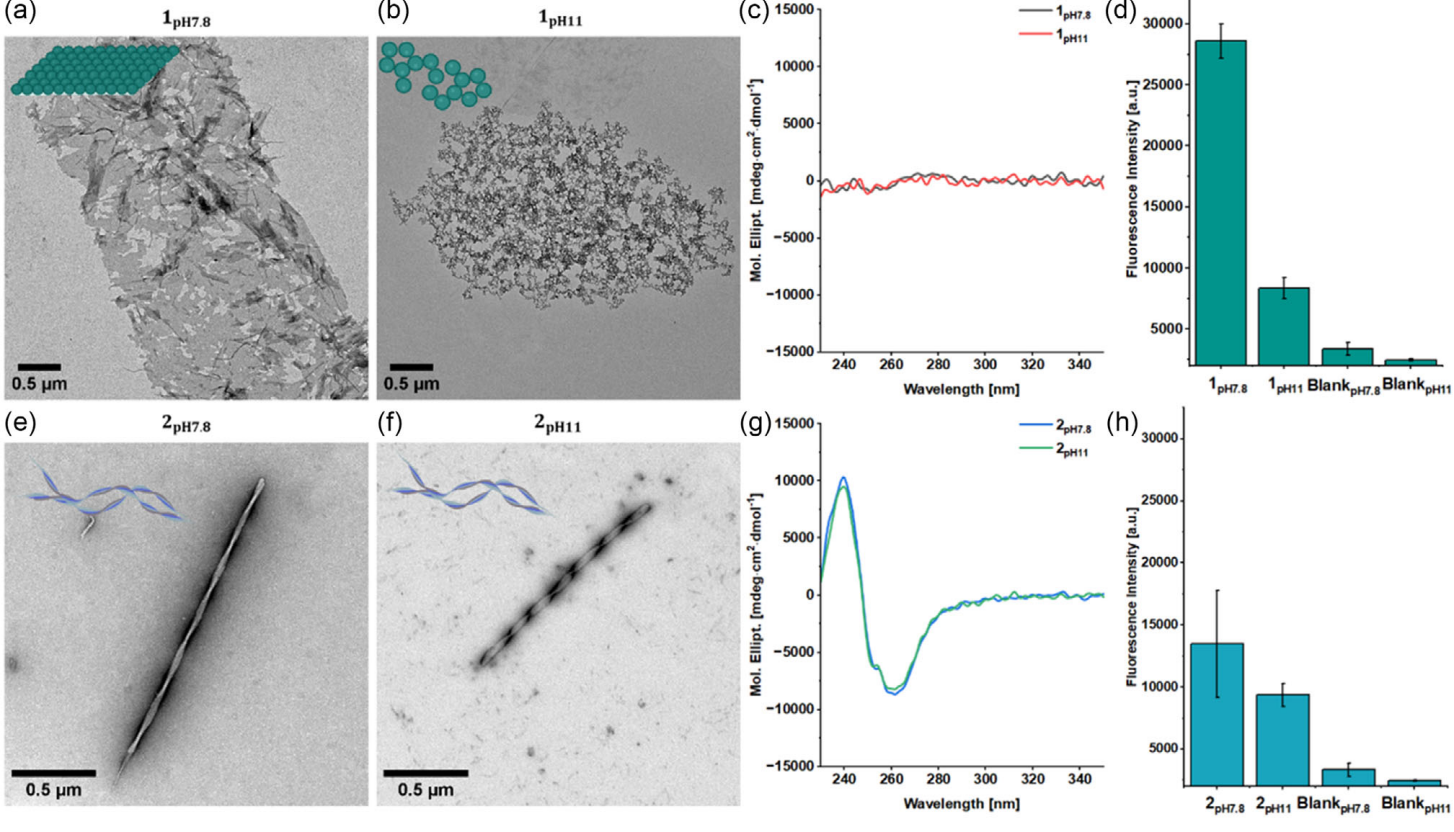
a) TEM image of $1_{pH7.8}$ revealing 2D‐sheets. b) TEM image of $1_{pH11}$ revealing unordered aggregates. c) CD spectra of $1_{pH7.8}$ and $1_{pH11}$ indicating that there is no coupling of the anthracene chromophores. d) Proteostat assay showing increased levels of *β*‐sheets for the sheets of $1_{pH7.8}$ compared to the aggregates of $1_{pH11}$. e) TEM image of $2_{pH7.8}$ revealing single twisted ribbons. f) TEM image of $2_{pH11}$ revealing single twisted ribbons. g) CD spectra of $2_{pH7.8}$ and $2_{pH11}$ indicating interactions of the anthracene according to the bisignate curve. h) Proteostat assay showing similar levels of *β*‐sheets in the nanostructures of $2_{pH7.8}$ and $2_{pH11}$. For the Proteostat assay, excitation wavelength of 550 nm and an emission wavelength of 600 nm were used. Blanks are negative controls without sample to account for background fluorescence. All TEM scale bars are 0.5 µm. Data presented as mean ± SEM (*n* = 3).

$1_{\mathrm{pH}7.8}$ was observed to form freestanding, nonuniform 2D‐sheets with irregular edges (**Figure** **2a**) whereas $1_{\mathrm{pH}11}$ did not assemble into ordered structures and formed only aggregates (Figure 2b). The increase in pH leads to a shift from an overall charge of 0 (pH 7.8), due to the zwitterion, to −1 (pH 11). This results in an enhanced electrostatic repulsion between the carboxylic acids of the C‐terminus, leading to the destabilization and the formation of aggregates instead.^[^ ^35^ ^]^ In comparison to **1**, **2** is extended by an alanine (A) and glutamic acid (E), resulting in an additional negative charge at pH 7.8 while preserving the alternating hydrophobic/hydrophilic pattern. The extended peptide sequence $2_{\mathrm{pH}7.8}$ assembles into single twisted ribbons. While the increase in pH to 11 increases the overall charge by −1, the same twisted ribbon morphology is retained for $2_{\mathrm{pH}11}$ (Figure 2e,f).

Although both peptides demonstrate an analogous increase in negative charge at elevated pH levels, the position of the lysine within the sequence varies. Specifically, peptide **1** features lysine positioned at the C‐terminus, exhibiting a pronounced alteration in morphology upon an increase in pH. Conversely, in peptide **2**, the lysine is positioned at the sequence's center and, therefore, there is no change in the local chemical environment at the peptide terminus and no change in morphology at elevated pH. This observation indicates that the local chemical environment at the C‐terminus, rather than the overall charge of the peptide, plays a pivotal role in determining its structural morphology.

The anthracene contribution toward the secondary structure was analyzed by CD spectroscopy from 230 nm onward as the presence of DMSO in the solvent composition shields the far UV region due to the absorbance of the S=O bond (<230 nm). As a result, signals corresponding to the peptide backbone's secondary structure, typically observed <220 nm, cannot be detected. $2_{\mathrm{pH}7.8}$ and $2_{\mathrm{pH}11}$ exhibit a bisignate curve with a negative cotton effect at 261 nm and a positive ellipticity at 240 nm, corresponding to the *π*‐π* transition of the anthracene. (Figure 2g). This observation suggests that the anthracenes are in close spatial proximity and possess a chiral orientation within the structure.^[^ ^36^, ^37^ ^–^ ^38^ ^]^ Conversely, $1_{\mathrm{pH}7.8}$ and $1_{\mathrm{pH}11}$ exhibit no coupling of the anthracene chromophores, indicating that the interactions between the aromatic groups are not the dominant driving force for assembly (Figure 2c).

To address the missing structural information pertaining to the peptide backbone, Fourier‐transform infrared spectroscopy (FTIR) and Proteostat assay were conducted. In addition, FTIR enables the visualization of the structural differences between the sheets of $1_{\mathrm{pH}7.8}$ and the twisted ribbons of $2_{\mathrm{pH}7.8}$. The spectrum of $1_{\mathrm{pH}7.8}$ exhibits a peak at 1625 cm^−1^, while $2_{\mathrm{pH}7.8}$ displays peaks at 1610 and 1625 cm^−1^ (Figure S19, Supporting Information). These peaks can be attributed to intermolecular *β*‐sheets, showing additional differences in secondary structure between $1_{\mathrm{pH}7.8}$ and $2_{\mathrm{pH}7.8}$.^[^ ^39^ ^,^ ^40^ ^]^ The *β*‐sheet content and molecular order are subsequently quantified with the fluorogenic Proteostat assay.^[^ ^31^ ^,^ ^41^ ^]^ $1_{\mathrm{pH}7.8}$ exhibited the highest fluorescence intensity, suggesting a high degree of order within the 2D, sheet‐like structures. In contrast, $1_{\mathrm{pH}11}$ which predominantly formed lower structural order aggregates exhibit reduced fluorescence intensity (Figure 2d). $2_{\mathrm{pH}7.8}$ and $2_{\mathrm{pH}11}$ showed comparable fluorescence intensities, which is in line with the CD and TEM results showing no difference in secondary structure or morphology upon increasing the pH (Figure 2h).

To establish the photochemistry of the anthracene units, we monitored the photodimerization reaction of peptides **1** and **2** using UV–vis spectroscopy, where the characteristic anthracene signal at 365 nm diminishes upon dimerization (Figure S9, Supporting Information). A quantitative framework for monitoring the dimerization is established by a calibration curve using different concentrations of anthracene to correlate the change in absorption and the remaining anthracene concentration (Figure S7, 8, Supporting Information). Using the calibration plot as a reference, the reactivity of both peptides (**1** and **2**) in their molecular forms (100 µM) was analyzed in a solvent mixture of 1:1 DMSO/NH_4_HCO_3_ buffer (10 mM) at pH 7.8 and pH 11 (adjusted with 1 M NaOH). Under these conditions, the monomers remain dissolved and no assembly takes place.

**Figure 3:**
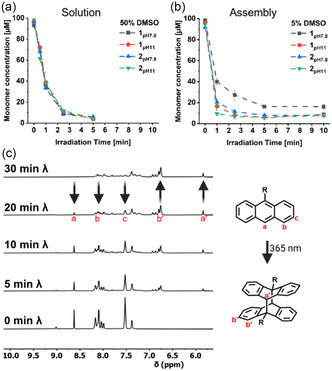
a) Consumption of the anthracene moiety of 1 and 2 in the nonassembled state with increasing irradiation time in 50/50 DMSO:Buffer (pH 7.8 or pH 11). b) Consumption of the anthracene moiety of 1 and 2 in the assembled state with increasing irradiation time in 5/95 DMSO:Buffer (pH 7.8 or pH 11). c) ^1^H‐NMR kinetic analysis of 1 at pH 7.8 at different irradiation times showing the decline in the anthracene signals while new signals of the dimer emerge. Data presented as mean ± SEM (*n* = 3).

In the molecular state, the monomer concentration was found to be less than 10% of its initial value after 5 min of 365 nm irradiation for peptides **1** and **2** across both pH values (**Figure** **3a**). Subsequently, the solvent composition was changed to 5:95 DMSO/NH_4_HCO_3_ buffer (10 mM) at pH 7.8 and pH 11 to initiate assembly, and the irradiation kinetics was repeated under the new conditions (Figure 3b). $1_{\mathrm{pH}7.8}$ exhibited slower kinetics compared to when it is dissolved in 50% DMSO, with substantial amount of monomer (16%) remaining even after 10 min irradiation. Conversely, $1_{\mathrm{pH}11}$ and $2_{\mathrm{pH}7.8}$ showed a lower remaining monomer concentration after 1 min and therefore a faster initial rate but still required 5 min to reach below 10%, similar to the irradiation in the nonassembled state. Peptide $2_{\mathrm{pH}11}$ demonstrated the most accelerated kinetics in the assembled state compared to its solution state, with less than 10% monomer remaining already after 1 min of 365‐nm irradiation (Figure 3b).

The collective secondary structure information revealed the kinetic discrepancies observed during irradiation. $1_{\mathrm{pH}7.8}$ demonstrates the highest degree of molecular order with no proximity coupling of the aromatic moieties, thereby preventing a substantial proportion of anthracene molecules from adopting the orientation needed for the dimerization reaction. For a successful photodimerization in the solid assembled state, the reaction centers need to be within ≈4.2 Å,^[^ ^42^ ^]^ and the lateral slip between the corresponding *π*‐orbitals to be less than ≈2 Å.^[^ ^43^ ^]^ If the molecular order within the assembly does not satisfy these requirements, the photochemical reaction slows considerably and prevents the complete conversion of the anthracene peptides. In contrast, the preorganization in $2_{\mathrm{pH}11}$ leads to an accelerated reaction, as the anthracenes are already in close spatial proximity within the assembly.

The [4 + 4] photodimerization was further confirmed by ^1^H‐NMR, which shows the emergence of a distinctive bridge proton signal at 5.8 ppm with the concomitant decrease of the respective anthracene signal at 8.6 ppm across all samples upon irradiation (Figure 3c). The bridge proton corresponds to the head‐to‐tail conformation of the dimer, which offers the least sterically hindered configuration, as evidenced by HMBC spectroscopy (Figure S14, Supporting Information). However, it was observed that only up to 60% conversion to the dimer was achieved, while the remaining monomer was found to be below 10% of the initial concentration (Figure S12, Supporting Information). While this result suggests the occurrence of side reactions upon irradiation, it is crucial to note that the overall concentration was 20‐fold higher than the assembly conditions to achieve a sufficient signal‐to‐noise ratio. Therefore, these experiments were conducted in a solution of 80% DMSO‐d_6_ and 20% NH_4_HCO_3_ buffer (10 mM) at pH 7.8 and pH 11, a composition that was found to ensure the solubility of all compounds at higher concentration while maintaining control over pH. The side reactions were investigated by using 9‐anthracen carboxylic acid as a model compound and revealed the emergence of 9,10‐anthraquinone upon irradiation besides the photodimer (Figure S15, Supporting Information). Additionally, it is important to note that the ratio between product and side reactions is dependent on the solvent composition (Figure S16, S17, Supporting Information). The difference in concentration and solvent composition can, in principle, alter the reactivity due to different mobility and electrostatic repulsion. The role of DMSO in this regard, compared to other organic co‐solvents such as THF, not only facilitates initial solubilization but also plays a role in modulating assembly morphology and photoreactivity.

**Figure 4:**
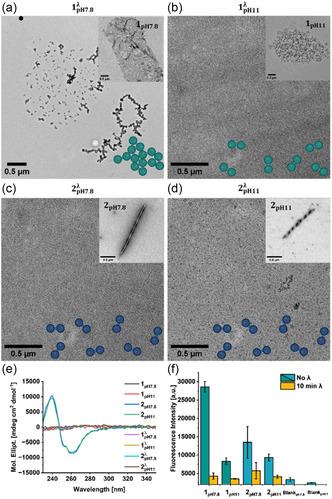
a) TEM image of $1_{pH7.8}^{\lambda }$ revealing unordered aggregates. Inset: TEM image of $1_{pH7.8}$ showing 2D‐sheets. b) TEM image of $1_{pH11}^{\lambda }$ revealing the absence of nanostructures. Inset: TEM image of $1_{pH11}$ showing unordered aggregates. c) TEM image of $2_{pH7.8}^{\lambda }$ revealing the absence of nanostructures. Inset: TEM image of $2_{pH7.8}$ showing twisted ribbons. d) TEM image of $2_{pH11}^{\lambda }$ revealing the absence of nanostructures. Inset: TEM image of $2_{pH11}$ showing twisted ribbons. e) CD spectra of 1 and 2 at pH 7.8 and 11 before and after irradiation. The CD spectra of the irradiated peptides $1_{pH11}^{\lambda }$, $1_{pH7.8}^{\lambda }$, $2_{pH7.8}^{\lambda }$ and $2_{pH11}^{\lambda }$ show no signal overall. f) Proteostat assay of 1 and 2 in different pH with and without irradiation at 365 nm showing the disassembly upon irradiation. For the Proteostat assay, excitation wavelength of 550 nm and an emission wavelength of 600 nm were used. Blanks are negative controls without sample to account for background fluorescence. All TEM scale bars are 0.5 µm. Data presented as mean ± SEM (*n* = 3).

We hypothesize that the different dimerization kinetics and efficiency as a consequence of molecular preorganization within the supramolecular structure can lead to different nanoscale outcomes. Using TEM, each nanostructure ($1_{\mathrm{pH}7.8}$, $1_{\mathrm{pH}11}$,$2_{\mathrm{pH}7.8}$,$2_{\mathrm{pH}11}$) was subjected to 10 min irradiation, a duration that has been shown to ensure maximum consumption of anthracene units (Figure 3b). In general, irradiated samples are denoted with *λ*. TEM analysis of $1_{\mathrm{pH}7.8}^{\lambda }$ revealed unordered aggregates, contrasting with the 2D‐sheets observed for the nonirradiated $1_{\mathrm{pH}7.8}$ (**Figure** **4a**). The dimer, with its bent conformation of the phenyl moieties, impedes the *π*‐*π* stacking of the planar aromatic groups, thereby causing a structural change toward aggregates. However, the reduction in attractive forces, as well as the geometric frustration induced by the dimerization, proves insufficient to cause full disassembly. Irradiation exhibits similar structural destabilization compared to pH increase, as evidenced by the unstructured aggregates observed in both the irradiated $1_{\mathrm{pH}7.8}^{\lambda }$ and the nonirradiated $1_{\mathrm{pH}11}$ at higher pH. The combination of pH and irradiation as destabilizing effects for $1_{\mathrm{pH}11}^{\lambda }$, results in a complete disassembly of the nanostructure, due to the decrease of *π*‐*π* stacking coupled with the increase in electrostatic repulsion (Figure 4b). The twisted ribbons of $2_{\mathrm{pH}7.8}$ exhibit a distinct response to irradiation as evidenced by the absence of both assemblies and aggregates in $2_{\mathrm{pH}7.8}^{\lambda }$(Figure 4c). This emphasizes the necessity of balancing hydrophobic and hydrophilic moieties to facilitate the dissolution of assembling units, as opposed to the formation of aggregates due to the collapse of hydrophobic regions, as **2** features a greater number of hydrophilic charged groups such as glutamic acids in comparison to **1**. Furthermore, the observation of disassembly in $2_{\mathrm{pH}11}^{\lambda }$ upon irradiation suggests that the morphology of the monomer and dimer assemblies of **2** is pH‐independent (Figure 4d). However, this process is solvent dependent, as irradiation of the assemblies of **2** in 1/99 DMSO/CHCl_3_ does not lead to disassembly (Figure S18, Supporting Information). The effect of irradiation on the supramolecular structure was further confirmed by CD and Proteostat assay. The CD signals for $2_{\mathrm{pH}7.8}^{\lambda }$ and $2_{\mathrm{pH}11}^{\lambda }$ are removed, thereby confirming the disassembly of the structure. $1_{\mathrm{pH}7.8}^{\lambda }$ and $1_{\mathrm{pH}11}^{\lambda }$ continue to demonstrate an absence of CD signal (Figure 4e). Proteostat assay confirmed these observations as the fluorescence intensity decreased upon irradiation in all cases (Figure 4f).

## 3 Conclusion

In summary, we have demonstrated the assembly of short anthracene‐containing peptides into different 1D and 2D morphologies by pH‐dependent charge modulation. Each morphology expresses different levels of molecular order and preorganization, leading to distinct kinetic responses toward the [4 + 4] photodimerization of anthracene units on the self‐assembling peptide. As a result, the extent of geometric frustration upon irradiation becomes selective toward nanostructures that promote proximity *π*‐*π* interactions of the anthracene moieties. Our results highlight that bimolecular photochemistry is a highly attractive avenue to integrate supramolecular selectivity into complex responsive systems, while offering the unique advantages of light. We believe that the technology will provide a new dimension toward light‐driven supramolecular materials and expand the toolbox for controlling disassembly processes.

## 4 Experimental Section

4.1

4.1.1

##### General Synthesis Procedure

The preloaded Wang resin was swollen in 5 mL DMF at room temperature for 1 h before transferring it into the peptide synthesizer. DMF was removed via a draining process, and the resin was swollen with 20 mL DMF for 20 s. Prior to each coupling step, as well as during the final deprotection, the N‐terminal Fmoc group was removed using two deprotection steps: 20% piperidine in DMF was applied for 15 s at 75 °C, followed by 50 s at 90 °C. Fmoc‐protected amino acids were coupled in a fivefold molar excess for 15 s and 110 s at 75 °C and 90 °C, respectively, using a solution of DIC (0.25 M) and Oxyma (0.5 M) in DMF. The solution was drained, and the resin washed with DMF three times. Anthracen was then coupled to the peptide by adding 9‐anthracene carboxylic acid (2 equiv.), PyBOP (2 equiv.) and DIPEA (4 equiv.) in DMF to the resin and shaking overnight. The peptide was cleaved from the resin by adding 10 mL of cleavage cocktail (95% TFA, 2.5% TIPS, 2.5% MilliQ water) to the resin and shaking at room temperature for 2 h. The cleavage cocktail was drained, and the resin was washed with TFA. The TFA containing solutions were combined, and the liquids were removed under reduced pressure.The peptide was purified via HPLC with a YMC‐Actus Triart C18 column (flow rate 20 mL min^−^^1^). After lyophilization, the peptide was received as a colorless solid.

##### General TEM Procedure

Peptide **1** or **2** (100 µM) was dissolved in 5% DMSO and 95% NH_4_HCO_3_ buffer (10 mM) and incubated overnight at 25 °C while shaking at 300 rpm. The solutions for irradiation were then transferred to a glass vial equipped with a stirring bar and covered with a blanket of argon. They were then irradiated at 365 nm for 10 min while stirring at 300 rpm and again incubated overnight at 25 °C. Then TEM grids were prepared by pipetting 3 µL of irradiated or nonirradiated peptide solution onto a Formvar‐coated copper grid and incubating for 5 min. After the incubation, the solutions were removed with filter paper, and the grids were stained with 7 µL 4% uranyl acetate solution for 2.5 min. The grids were washed three times with MilliQ water and dried before being measured.

## Conflicts of Interest

The authors declares no conflicts of interest.

## Supporting information

Supplementary Material

## Data Availability

The data that support the findings of this study are available in the supplementary material of this article.
