# Supplementary material for: Light‐Triggered Disassembly of Peptide Nanostructures
Source: Chembiochem. 2025 Aug 14;26(16):e202500414. doi: 10.1002/cbic.202500414 (PMC12432488; doi:10.1002/cbic.202500414)
Supplement: Supplementary file 1 — Supplementary Material [file CBIC-26-e202500414-s001.pdf]

Supporting Information

Light-Triggered Disassembly of Peptide Nanostructures

Raphael Meyer,[a] Julian Link,[a] Lucas Gunkel,[b] Albin Lahu,[a] Hakan Demirezen,[a] Tanja Weil[a] and David Y. W. Ng*[a]

[a] R. Meyer, J.Link, A.Lahu, H.Demirezen, Prof. T. Weil, Dr. D.Y.W. Ng
Department of the Synthesis of Macromolecule
Max Planck Institute for Polymer Research
Ackermannweg 10, Mainz 55128, Germany
E-mail: david.ng@mpip-mainz.mpg.de, weil@mpip-mainz.mpg.de

[b] L. Gunkel
Department of Molecular Spectroscopy
Max Planck Institute for Polymer Research
Ackermannweg 10, Mainz 55128, Germany

Table of Contents

1 Materials and Instruments 3
1.1 Materials 3
1.2 Instruments 3
1.2.1 Microwave Peptide Synthesizer 3
1.2.2 High-Performance Liquid Chromatography (HPLC) 3
1.2.2 Nuclear Magnetic Resonance Spectroscopy (NMR) 3
1.2.3 1.2.4 Liquid Chromatography – Mass Spectrometry (LC-MS) 3
1.2.4 Circular Dichroism Spectroscopy (CD) 4
1.2.5 Fluorescence Spectroscopy 4
1.2.6 Transmission Electron Microscopy (TEM) 4
1.2.7 Irradiation setup 4
1.2.8 Fourier-transform Infrared spectroscopy 4
2 Synthesis 5
2.1 Ant-GISVK 5
2.2 Ant-GISVKAE 7
3 Characterization 10
3.1 Absorbance and Fluorescence spectroscopy 10
3.1.1 Calibration curves 10
3.1.2 Irradiation conversion in 50% DMSO 11
3.1.3 Irradiation conversion in 5% DMSO 12
3.1.4 Proteostat assay 12
3.2 Nuclear magnetic resonance 13
3.3 Transmission electron microscopy 19
3.3.1 Non-irradiated samples 19
3.3.2 Irradiated samples 19
3.4 Circular Dichroism 20
3.5 Fourier-transform Infrared spectroscopy 21

1 Materials and Instruments

1.1 Materials

All chemicals were purchased from commercial sources and were used without further purification. Solvents and reagents used for peptide synthesis were obtained in peptide grade. Purification with HPLC was performed with water obtained from a Millipore purification system and HPLC grade acetonitrile.

1.2 Instruments

1.2.1 Microwave Peptide Synthesizer

Peptides and peptide fractions were synthesized with a Liberty Blue Automated Microwave Peptide Synthesizer by CEM Corporation.

1.2.2 High-Performance Liquid Chromatography (HPLC)

The synthesized peptides were purified by preparative HPLC using a Shimadzu setup. A YMC-Actus Triart C18 column (150 x 20 mm, 5 µm) with a flowrate of 20 mL/min or a Phenomenex Kinetex EVO C18 100 (150 x 30 mm, 5 µm) with a flowrate of 25 mL/min was used for purification.
All purification steps were performed by using gradients of MilliQ water and acetonitrile, each acidified with 0.1% formic acid. Analytical measurements were performed by using gradients of MilliQ water and acetonitrile, each acidified with 0.1% trifluoroacetic acid. The HPLC data were processed with the software LabSolutions by Shimadzu and OriginPro 2024b by OriginLab.

1.2.2 Nuclear Magnetic Resonance Spectroscopy (NMR)

NMR spectra were recorded on a Bruker Avance III 700 MHz NMR spectrometer. For ^{1}H spectra, the chemical shifts are reported in parts per million (ppm) from high to low frequency using the residual solvent peak as the internal reference (DMSO-d_6 = 2.50 (C**H**D_2SOCD_3) ppm). All ^{1}H resonances are reported to the nearest 0.01 ppm. The multiplicity of ^{1}H signals are indicated as: s = singlet; d = doublet; t = triplet; q = quartet; p = pentet; m = multiplet; br = broad; or combinations of thereof. Coupling constants (*J*) are quoted in Hz and reported to the nearest 0.1 Hz. Where appropriate, averages of the signals from peaks displaying multiplicity were used to calculate the value of the coupling constant. The data was processed in MestReNova.

1.2.3 1.2.4 Liquid Chromatography – Mass Spectrometry (LC-MS)

Peptides were analysed by HPLC-ESI-MS on a LC-MS 2020 setup by Shimadzu using a Kinetex EVO C18 100 Å LC column (50 × 2.1 mm, 2.6 µm). Acetonitrile (ACN) and MilliQ water acidified with 0.1% formic acid were used as solvents for all measurements. The solvent gradient

started with 5% ACN and 95% water, while the ACN content was linearly increased to 95% in 12 min. Data were processed in LabSolutions and OriginPro 2024b by OriginLab.

1.2.4 Circular Dichroism Spectroscopy (CD)

CD spectra of the peptides were recorded on a JASCO J-1500 spectrometer in a 0.1 cm High Precision Cell by HellmaAnalytics. The recorded data were processed in Spectra Analysis by JASCO and OriginPro 2024b by OriginLab.

1.2.5 Fluorescence Spectroscopy

To record the fluorescence intensity, a SPARK 20M microplate reader by Tecan Group Ltd. was used. The samples were measured in a Greiner 384 flat black well plate and data was processed in OriginPro 2024b by OriginLab. The absorbance was recorded on the same instrument with a Greiner UV-STAR® plate, 384 well, F-Bottom, clear.

1.2.6 Transmission Electron Microscopy (TEM)

TEM images of the peptide samples were recorded on a JEOL 1400 transmission electron microscope at a voltage of 120 kV. Formvar/carbon-film coated copper grids (300 mesh) by Plano GmbH were used to prepare the samples. The images were processed in Fiji ImageJ.

1.2.7 Irradiation setup

Irradiations were performed with a Thorlabs M365L3 lamp with a nominal wavelength of 365 nm, a bandwith (FWHM) of 9 nm and a current of 1000 mA.

1.2.8 Fourier-transform Infrared spectroscopy

Infrared spectra were recorded in transmission geometry using a Bruker Vertex 70 IR spectrometer. The spectrometer has a resolution of 4 cm^{-1} in a range from 400 cm^{-1} to 4000 cm^{-1}.

2 Synthesis

2.1 Ant-GISVK

The Wang resin preloaded with Fmoc-Lys(Boc) (0.1 mmol) was swollen in 5 mL DMF at room temperature for 1 h before transferring it into the peptide synthesizer. DMF was removed via a draining process and the resin swollen with 20 mL DMF for 20 s. Prior to each coupling step, as well as during the final deprotection, the N-terminal Fmoc group was removed using two deprotection steps: 20% piperidine in DMF (3 ml) was applied for 15 seconds at 75 °C, followed by 50 seconds at 90 °C. Fmoc-protected amino acids (Fmoc-Val, Fmoc-Ser(tBu), Fmoc-Ile and Fmoc-Gly) were coupled in a 5-fold molar excess (5 eq. in 1.25 ml) for 15 seconds and 110 seconds at 75 °C and 90 °C, respectively, using a solution of DIC (0.25 M) and Oxyma (0.5 M) in 2.75 ml DMF. The solution was drained, and the resin washed with DMF three times. Anthracen was then coupled to the peptide by adding 9-anthracene carboxylic acid (44.45 mg, 2 equiv.), PyBOP (104 mg, 2 equiv.) and DIPEA (68 µL, 4 equiv.) in 5 mL DMF to the resin and shaking overnight. The peptide was cleaved from the resin by adding 10 mL of cleavage cocktail (95% TFA, 2.5% TIPS, 2.5% MilliQ water) to the resin and shaking at room temperature for 2 h. The cleavage cocktail was drained, and the resin washed with TFA. The TFA containing solutions were combined and the liquids removed under reduced pressure.
The peptide was purified via HPLC with a YMC-Actus Triart C18 column (flowrate 20 mL/min). After lyophilisation, the product Ant-GISVK was received as a colourless solid (14.13 mg, 0.02 mmol, 20%).

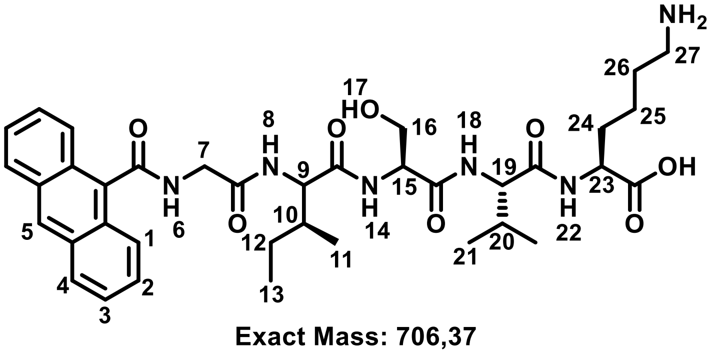

^{1}H-NMR (700 MHz, DMSO-d$_6$) δ [ppm] = 9.05 (t, J = 6.1 Hz, 2H, **6**), 8.65 (s, 1H, **5**), 8.25 (d, J = 36.1 Hz, 2H, **1**), 8.13–8.10 (m, 3H, **4,8**), 8.08 (d, J = 7.6 Hz, 1H, **14**), 8.03 (d, J = 8.2 Hz, 1H, **18**), 7.58–7.51 (m, 4H, **2,3**), 7.48 (d, J = 7.1 Hz, 1H, **22**), 4.51 (q, J = 6.6 Hz, 1H, **15**), 4.46 (dd, J = 9.0, 7.6 Hz, 1H, **9**), 4.13 (d, J = 49.6, 16.1, 6.0 Hz, 2H, **7**), 4.05 (dd, J = 8.2, 5.4Hz, 1H, **19**), 3.83 (q, J = 6.2 Hz, 1H, **23**), 3.63 (qd, J = 10.7, 6.1 Hz, 2H, **16**), 2.71 (t, J = 9.6 Hz, 2H, **27**), 2.12 m(dd, J = 13.0, 6.5 Hz, 1H, **20**), 1.79–1.73 (d, 1H, **10**), 1.59 (d, J = 8.9 Hz, 1H, **26**), 1.51 (dd, J = 14.4, 7.4 Hz, 4H, **12, 24, 26**), 1.36–1.19 (m, 2H, **25**), 0.910.82 (m, 12H, **11, 13, 21**).

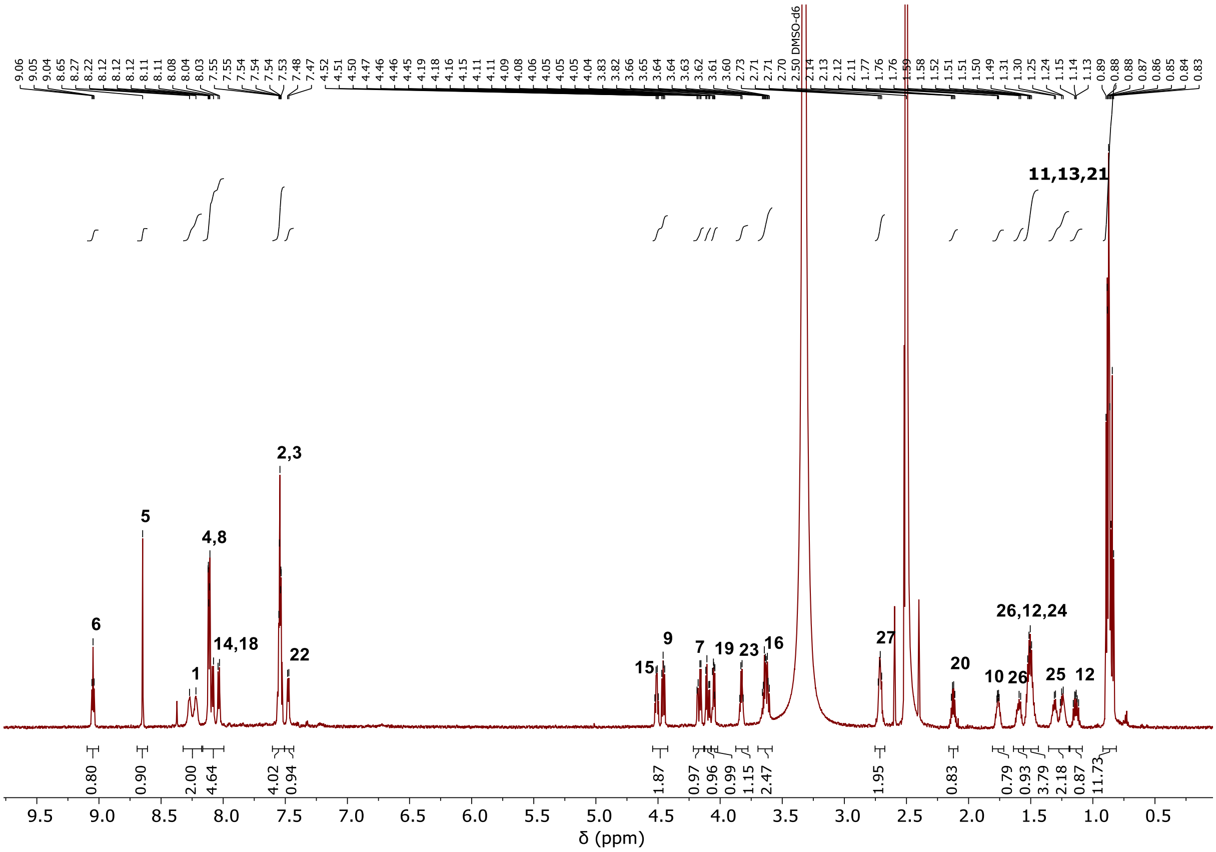

Figure 1: ^{1}H NMR spectrum (700 MHz, DMSO-d_6, 298 K) of compound **1**

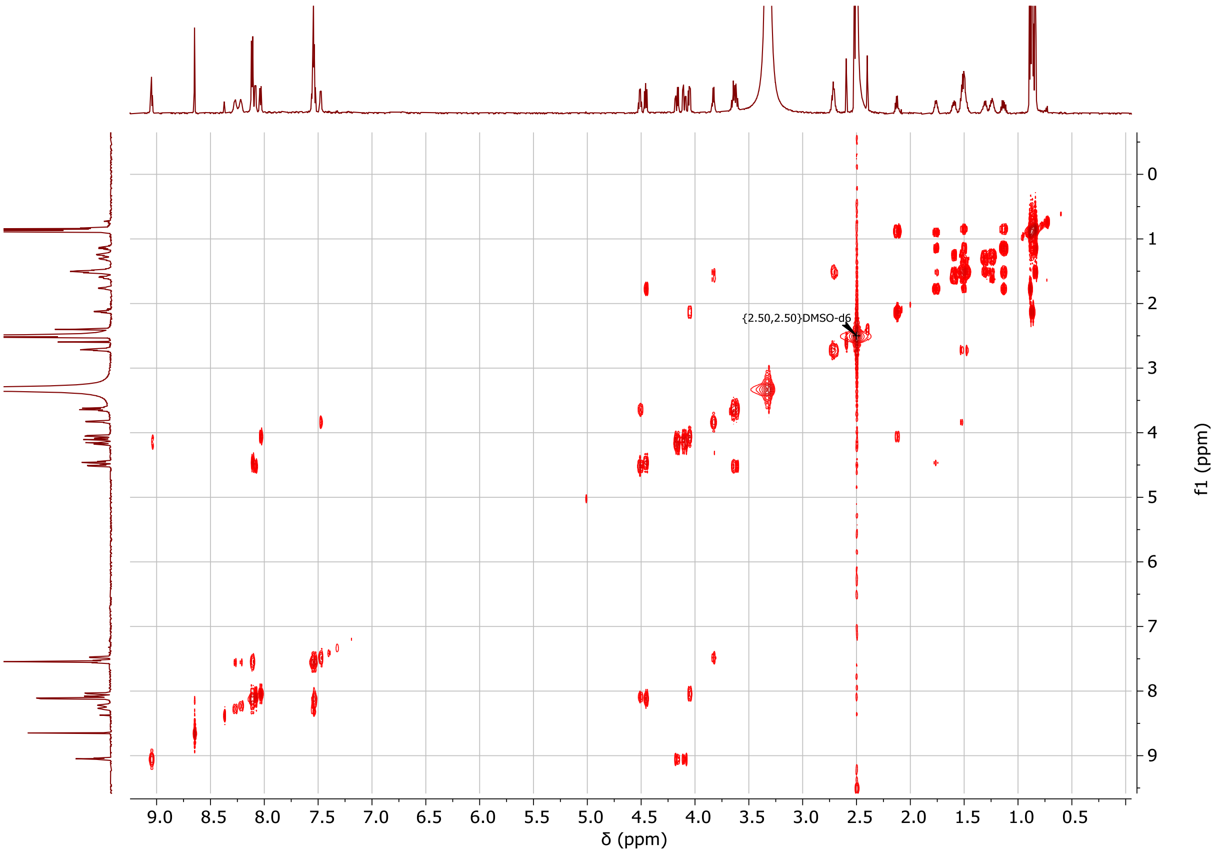

Figure 2: ^{1}H NMR and $^{1}H,^{1}H$ COSY NMR spectra (700 MHz, DMSO-d_6, 298 K) of compound **1**

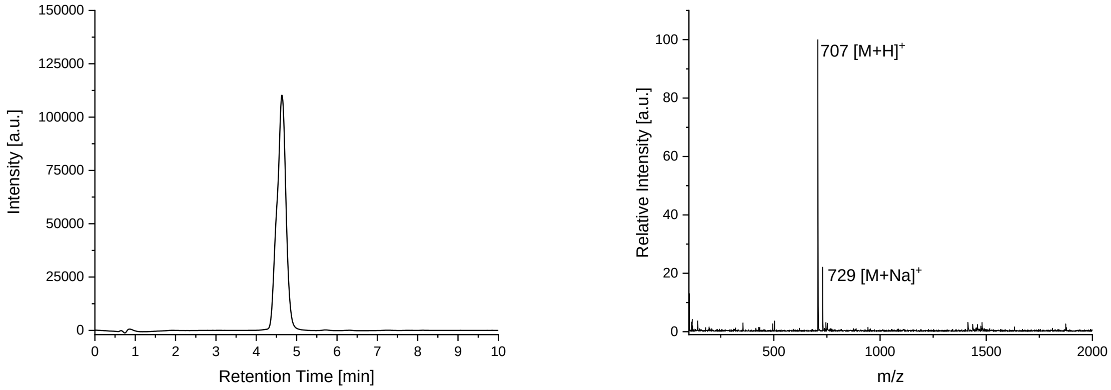

Figure 3: LC spectrum (left) and ESI MS (positive mode, right) of **1**. m/z calculated: $[M+H]^+$ = 707, found: $[M+H]^+$ = 707, $[M+Na]^+$ = 729.

2.2 Ant-GISVKAE

The Wang resin preloaded with Fmoc-Glu(OtBu) (0.25 mmol) was swollen in 5 mL DMF at room temperature for 1 h before transferring it into the peptide synthesizer. DMF was removed via a draining process and the resin swollen with 20 mL DMF for 20 s. Prior to each coupling step, as well as during the final deprotection, the N-terminal Fmoc group was removed using two deprotection steps: 20% piperidine in DMF (3 ml) was applied for 15 seconds at 75 °C, followed by 50 seconds at 90 °C. Fmoc-protected amino acids (Fmoc-Ala, Fmoc-Lys(Boc), Fmoc-Val, Fmoc-Ser(tBu), Fmoc-Ile and Fmoc-Gly) were coupled in a 5-fold molar excess (5 eq. in 1.25 ml) for 15 seconds and 110 seconds at 75 °C and 90 °C, respectively, using a solution of DIC (0.25 M) and Oxyma (0.5 M) in 2.75 ml DMF. The solution was drained, and the resin washed with DMF three times. Anthracen was then coupled to the peptide by adding 9-anthracene carboxylic acid (111 mg, 2 equiv.), PyBOP (260 mg, 2 equiv.) and DIPEA (170 µL, 4 equiv.) in 5 mL DMF to the resin and shaking overnight. The peptide was cleaved from the resin by adding 10 mL of cleavage cocktail (95% TFA, 2.5% TIPS, 2.5% MilliQ water) to the resin and shaking at room temperature for 2 h. The cleavage cocktail was drained, and the resin washed with TFA. The TFA containing solutions were combined and the liquids removed under reduced pressure.
The peptide was purified via HPLC with a YMC-Actus Triart C18 column (flowrate 20 mL/min). After lyophilisation, the product Ant-GISVKAE was received as a colourless solid (50.6 mg, 0.06 mmol, 24%).

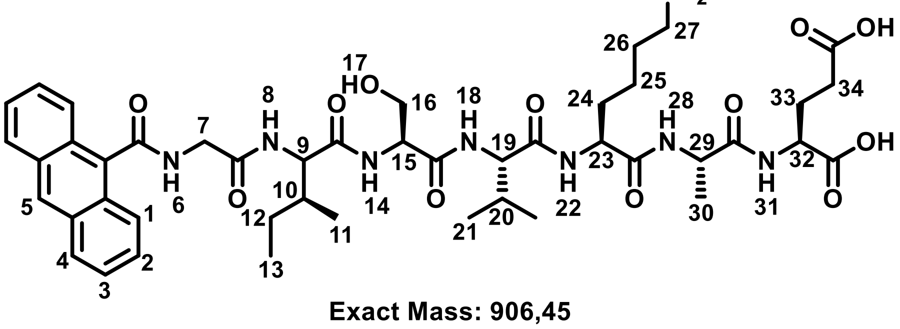

^{1}H NMR (700 MHz, DMSO) δ 9.02 (t, *J* = 6.2 Hz, 1H, **6**), 8.65 (s, 1H, **5**), 8.53 (d, *J* = 7.6 Hz, 1H, **28**), 8.27 (d, *J* = 7.7 Hz, 2H, **1**), 8.23 (s, 1H, **14**), 8.14 – 8.10 (m, 2H, **4**), 8.08 (d, *J* = 9.0 Hz, 1H, **8**), 8.04 (d, *J* = 7.7 Hz, 1H, **31**), 7.69 (d, *J* = 8.8 Hz, 1H, **18**), 7.54 (dd, *J* = 7.1, 3.0 Hz, 4H, **2**, **3**), 7.33 (d, *J* = 6.5 Hz, 1H, **22**), 4.47 (t, *J* = 8.0 Hz, 1H, **9**), 4.41 (q, *J* = 6.2 Hz, 1H, **15**), 4.29 (q, *J* = 7.6 Hz, 1H, **32**), 4.24 – 4.05 (m, 4H, **7, 29, 19**), 3.81 (q, *J* = 6.4 Hz, 1H, **23**), 3.59 (d, *J* = 6.1 Hz, 3H, **16**), 2.83 – 2.66 (m, 2H, **34**), 2.26 – 2.06 (m, 2H, **27**), 1.96 (q, *J* = 6.8 Hz, 1H, **20**), 1.85 – 1.65 (m, 4H, **26'**, **24**, **10**), 1.60 – 1.27 (m, 6H, **26'**, **33**, **12'**, **25**), 1.22 (d, *J* = 7.3 Hz, 3H, **30**), 1.14 (dt, *J* = 14.0, 7.7 Hz, 1H, **12'**), 0.98 – 0.67 (m, 12H, **11**, **13**, **21**).

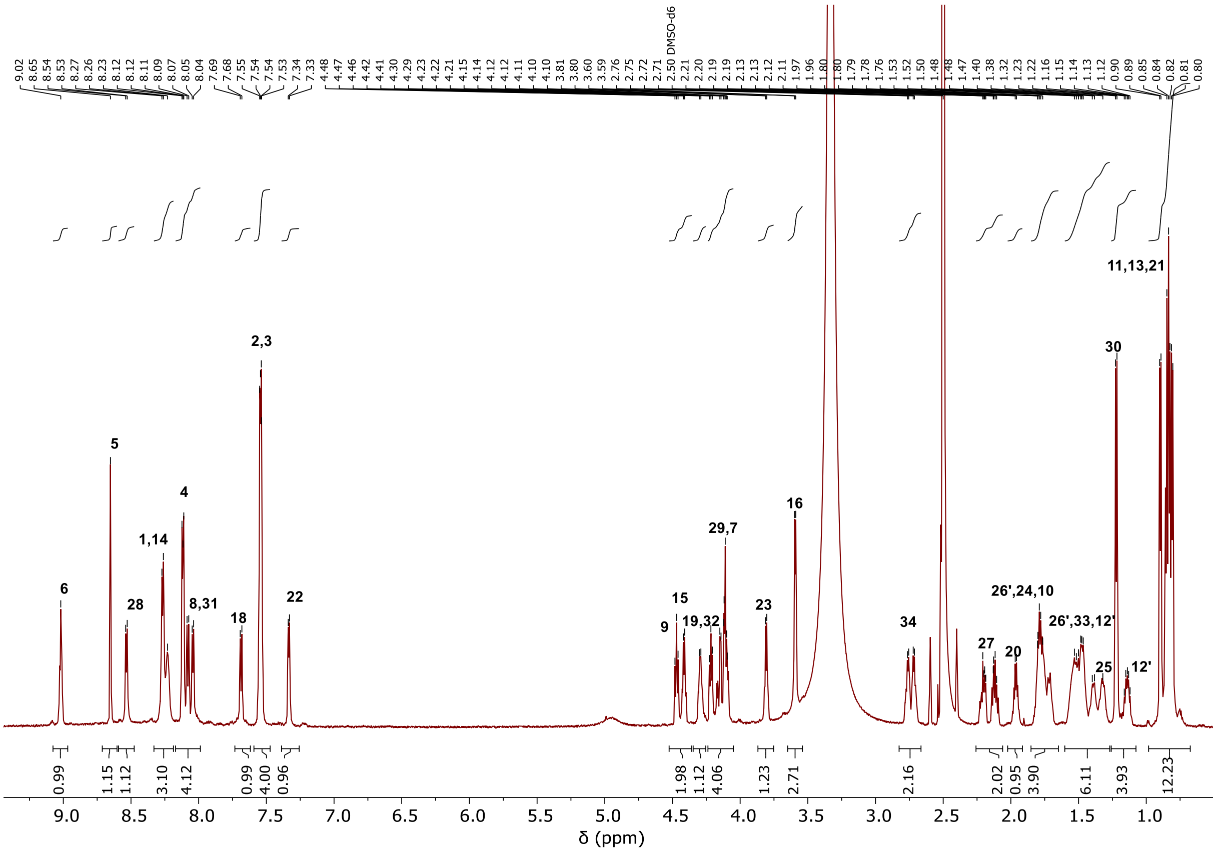

Figure 4: ^{1}H NMR spectrum (700 MHz, DMSO-d6, 298 K) of compound **2**

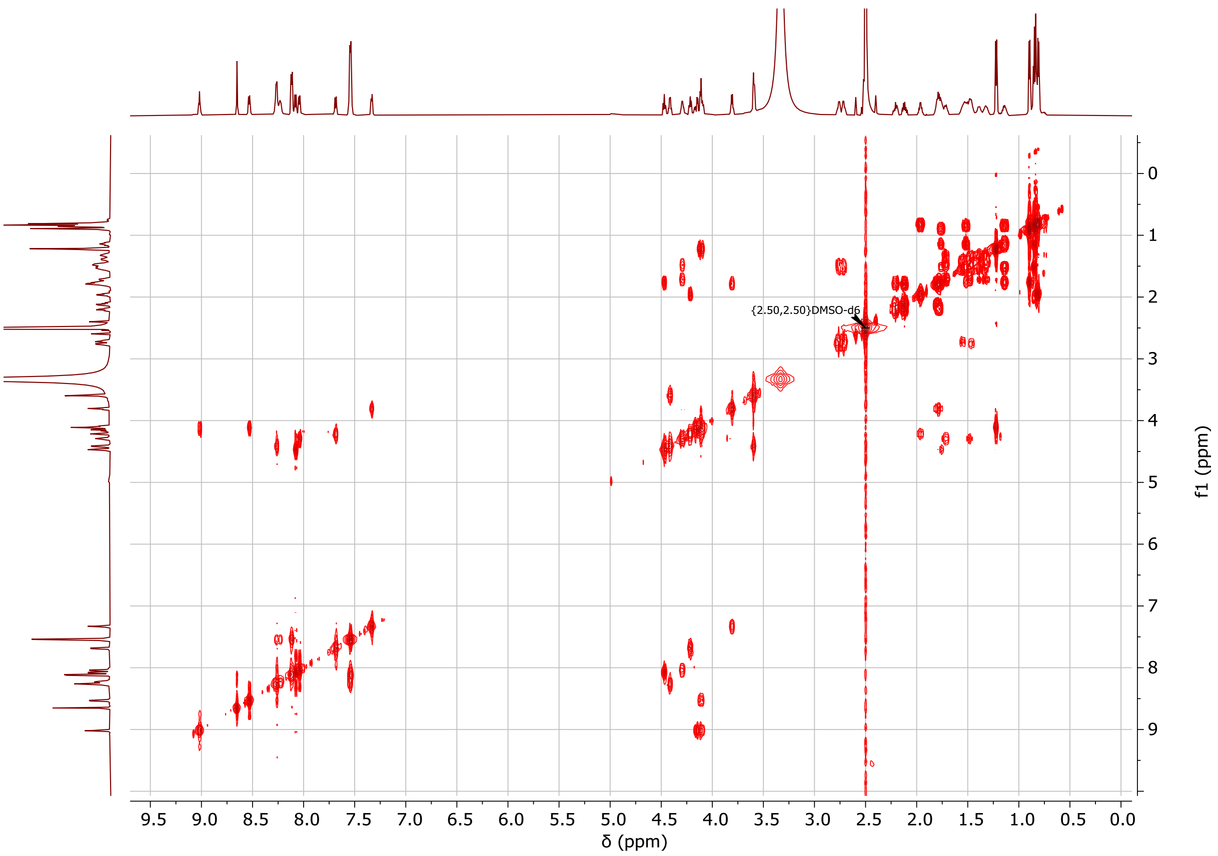

Figure 5: ^{1}H NMR and $^{1}H,^{1}H$ COSY NMR spectra (700 MHz, DMSO-d_6, 298 K) of compound **2**

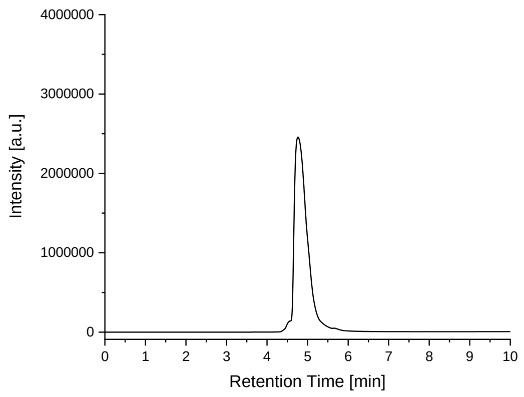

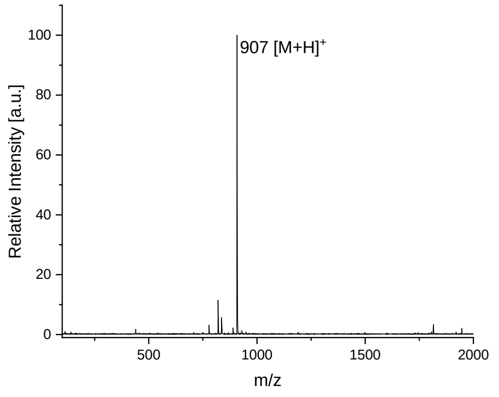

Figure 6: LC spectrum (left) and ESI MS (positive mode, right) of **2**. m/z calculated: $[M+H]^+$ = 907, found: $[M+H]^+$ = 907.

3 Characterization

3.1 Absorbance and Fluorescence spectroscopy

3.1.1 Calibration curves

1 was dissolved in 50% DMSO and 50% NH_4HCO_3 buffer (10 mM, pH 7.8) in the following concentrations: 100, 50, 25, 12.5, 6.25 and 0 µM. Then for each concentration three times 60 µL were transferred to a Greiner 384 UV-STAR® clear wellplate with flat bottom and the absorbance at 366 nm was measured. Absorbance was then plotted against the concentration and the points fitted linearly. The same procedure was conducted for **2**.

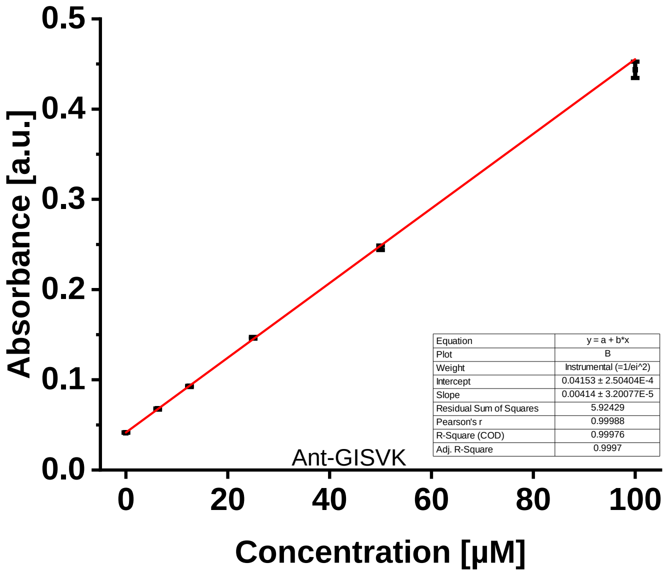

Figure 7: Linear fit of the absorbances of **1** at 366 nm with different concentrations in 50% DMSO and 50% NH_4HCO_3 buffer (10 mM, pH 7.8).

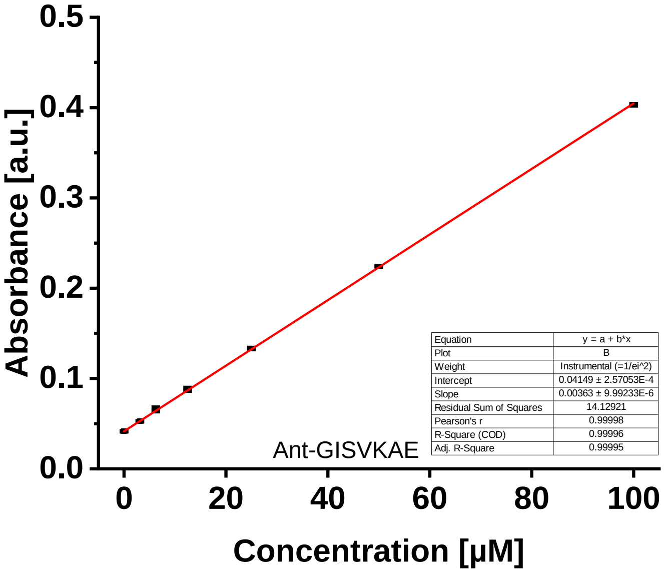

Figure 8: Linear fit of the absorbances of **2** at 366 nm with different concentrations in 50% DMSO and 50% NH_4HCO_3 buffer (10 mM, pH 7.8).

3.1.2 Irradiation conversion in 50% DMSO

1 (100 µM) was dissolved in 500 µL of 50% DMSO and 50% NH_4HCO_3 buffer (10 mM, pH 7.8) in a glass vial equipped with a stirring bar and covered with a blanket of argon. The solution was then irradiated at 365 nm for 0.5, 1, 2.5 and 5 min while stirring at 300 rpm. After each irradiation timepoint the absorbance at 366 nm was measured with three times 60 µL using a Tecan plate reader and Greiner 384 UV-STAR® clear wellplates with flat bottom. After each measurement the solution was filled back to the glass vial to keep the volume consistent.

This procedure was repeated for **1** in 50% DMSO and 50% NH_4HCO_3 buffer (10 mM, pH 11 (adjusted with 1 M NaOH solution)) and for **2** at both pH values.

The concentration of remaining **1** or **2** was then calculated with the absorbance values using the calibration curves of **3.1.1.**

Additionally UV-Vis spectra between 250 nm and 400 nm were recorded of each peptide at both pH values before and after 5 min of irradiation at 365 nm.

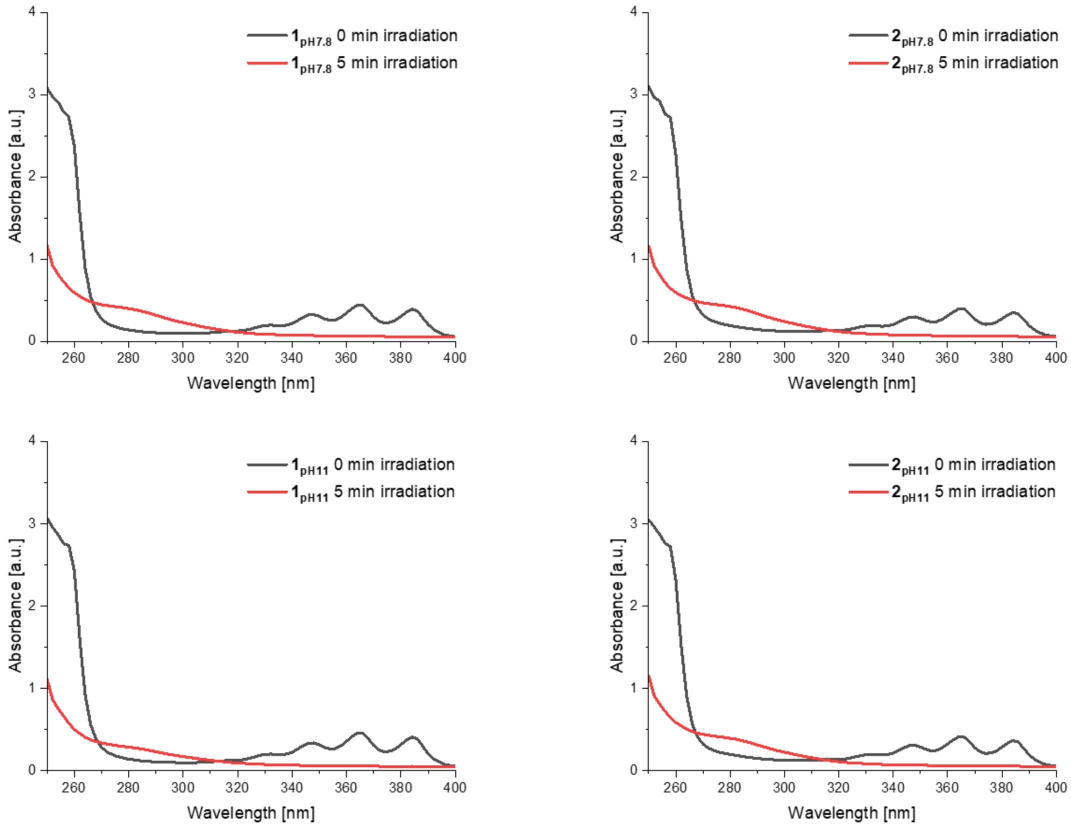

Figure 9: UV-VIS spectra of peptides **1** and **2** in 50/50 DMSO:Buffer (pH 7.8 or pH 11) after 0 or 5 min irradiation at 365 nm.

3.1.3 Irradiation conversion in 5% DMSO

1 (100 µM) was dissolved in 150 µL of 5% DMSO and 95% NH_4HCO_3 buffer (10 mM, pH 7.8) and incubated overnight at 25°C while shaking at 300 rpm. The solution was then transferred to a glass vial equipped with a stirring bar and covered with a blanket of argon. The solution was then irradiated at 365 nm for 1 min while stirring at 300 rpm and then diluted with 135 µL DMSO to achieve a DMSO content of 50%. The absorbance at 366 nm was measured with three times 60 µL using a Tecan plate reader and Greiner 384 UV-STAR® clear wellplates with flat bottom. This process was repeated for irradiation times of 0, 2.5, 5 and 10 min of irradiation at 365 nm.

The procedure was conducted for **1** in 5% DMSO and 95% NH_4HCO_3 buffer (10 mM, pH 11) and for **2** at both pH values.

The concentration of remaining **1** or **2** was then calculated with the absorbance values using the calibration curves of **3.1.1** taking into account the dilution with DMSO.

3.1.4 Proteostat assay

1 (100 µM) was dissolved in 5% DMSO and 95% NH_4HCO_3 buffer (10 mM, pH 7.8) and incubated overnight at 25°C while shaking at 300 rpm.

The Proteostat Protein Aggregation Assay Kit was purchased from *Enzo Life Sciences, Inc*. 0.52 µL of the Proteostat stock solution was diluted with 98.48 µL MilliQ water and 1 µL assay

buffer. 1 µL of this solution was added to 9 µL peptide solution in a Greiner 384 flat black wellplate three times for each sample. The solutions were incubated in the dark for 15 min while shaking at 500 rpm. The fluorescence intensity was measured at an excitation wavelength of 550 nm and emission wavelength of 600 nm and with a bandwidth of 20 nm. The procedure was conducted for **1** in 5% DMSO and 95% NH_4HCO_3 buffer (10 mM, pH 11) and for **2** at both pH values.
Samples for irradiation were transferred to a glass vial equipped with a stirring bar and covered with a blanket of argon. They were each irradiated at 365 nm for 10 min while stirring at 300 rpm and again incubated overnight at 25°C.

3.2 Nuclear magnetic resonance

1.4 mg of **1** was dissolved in 600 µL DMSO-d_6 and 150 µL NH_4HCO_3 buffer in H_2O (10 mM, pH 7.8). ^{1}H-NMR was then measured with water suppression. The sample was then covered with a blanket of argon and irradiated for 5 min at 365 nm while stirring at 300 rpm. ^{1}H-NMR with water suppression was again measured and this process repeated for a total of 10, 20 and 30 min of irradiation at 365 nm. The integral of the 1H-singulett of the anthracene monomer at 8.63 ppm and of the anthracene-dimer at 5.84 ppm were compared to each other while using the peptide signal at 2.69 ppm as a standard.
The procedure was conducted for **1** in 80% DMSO-d_6 and 20% NH_4HCO_3 buffer in H_2O (10 mM, pH 11) and for **2** at both pH values.

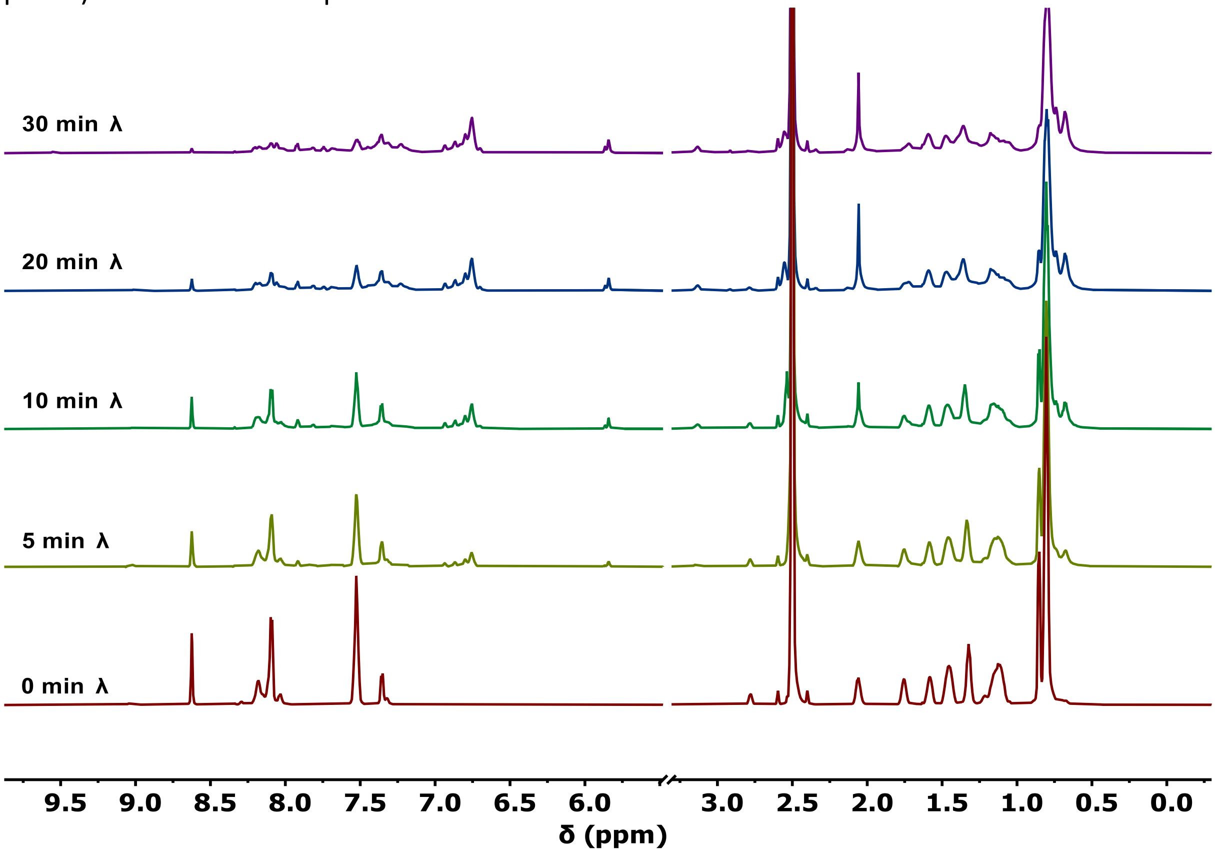

Figure 10: ^{1}H NMR kinetic (700 MHz, 298 K) of **1** at different irradiation times in 80% DMSO-d_6 and 20% NH_4HCO_3 buffer in H_2O (10 mM, pH 11).

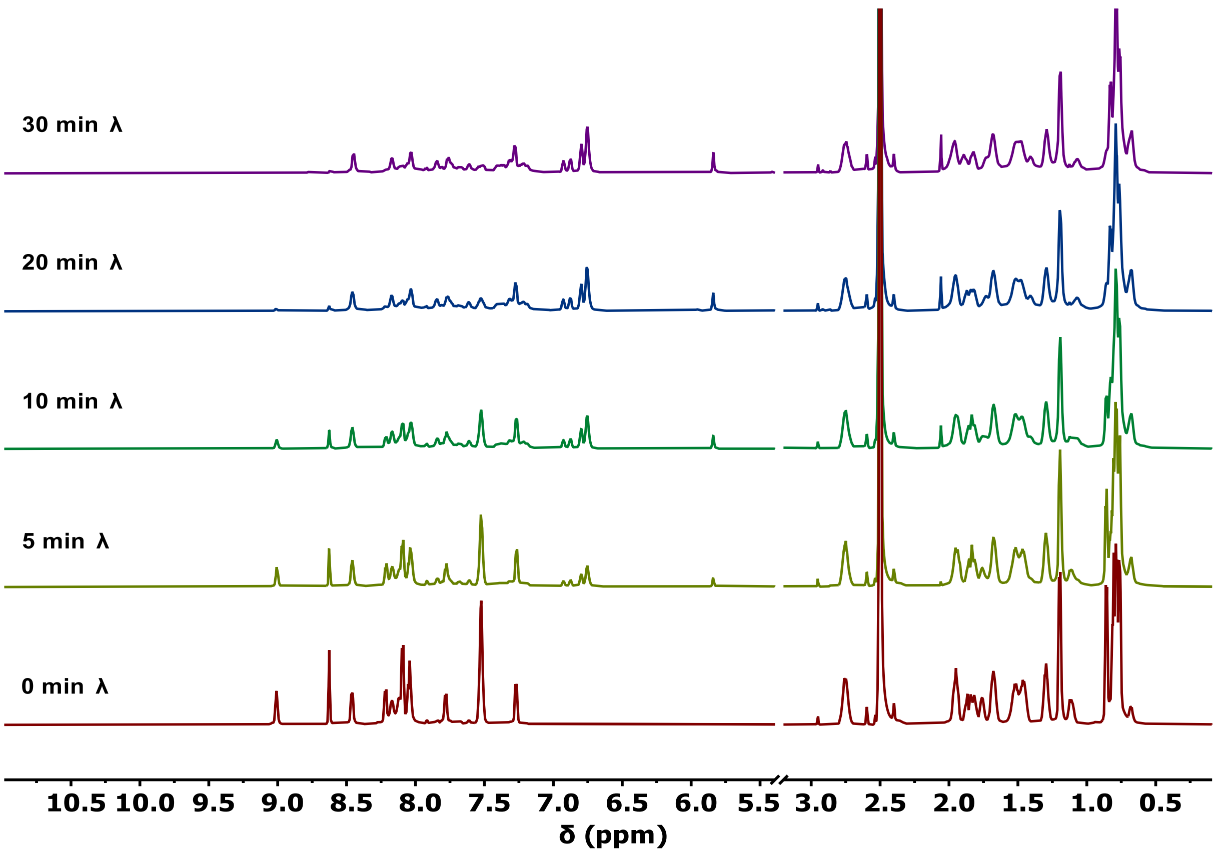

Figure 11: ^{1}H NMR kinetic (700 MHz, 298 K) of **2** at different irradiation times in 80% DMSO-d_6 and 20% NH_4HCO_3 buffer in H_2O (10 mM, pH 7.8).

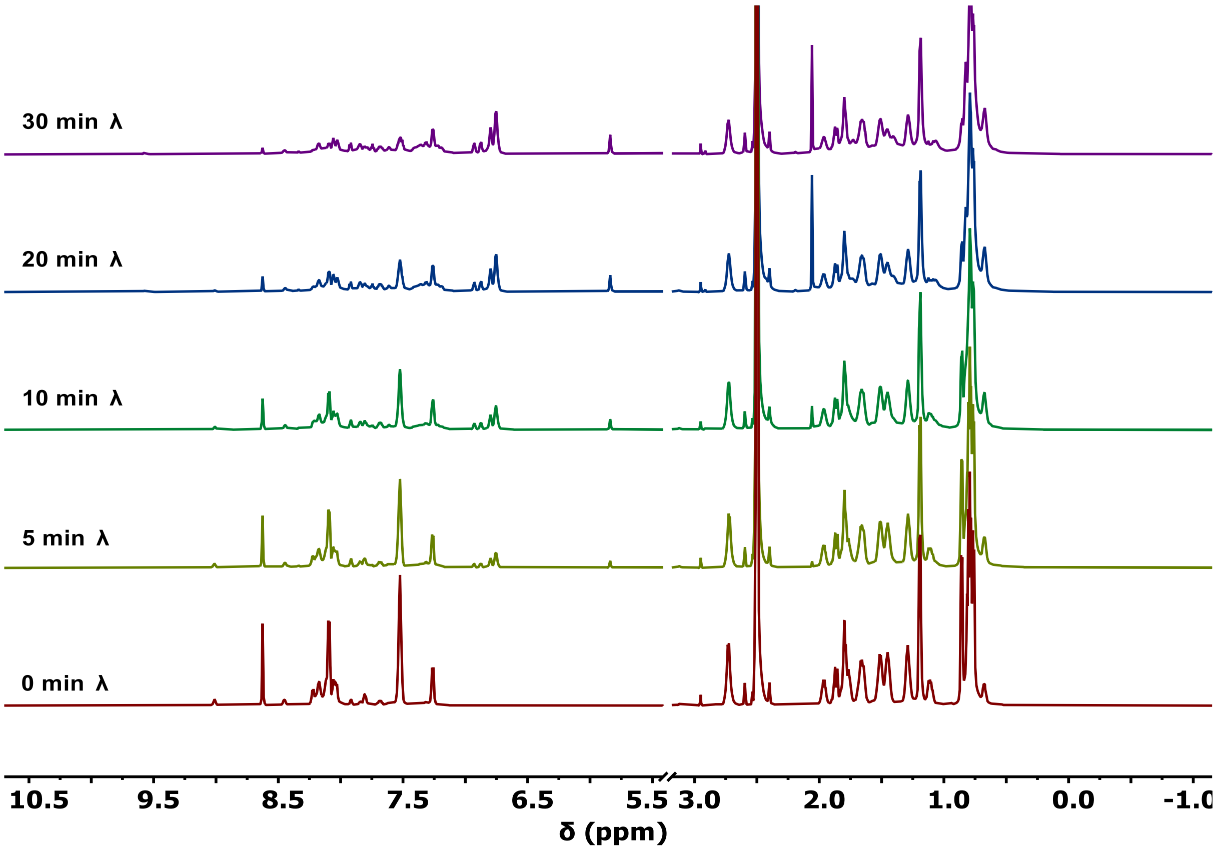

Figure 12: ^{1}H NMR kinetic (700 MHz, 298 K) of **2** at different irradiation times in 80% DMSO-d_6 and 20% NH_4HCO_3 buffer in H_2O (10 mM, pH 11).

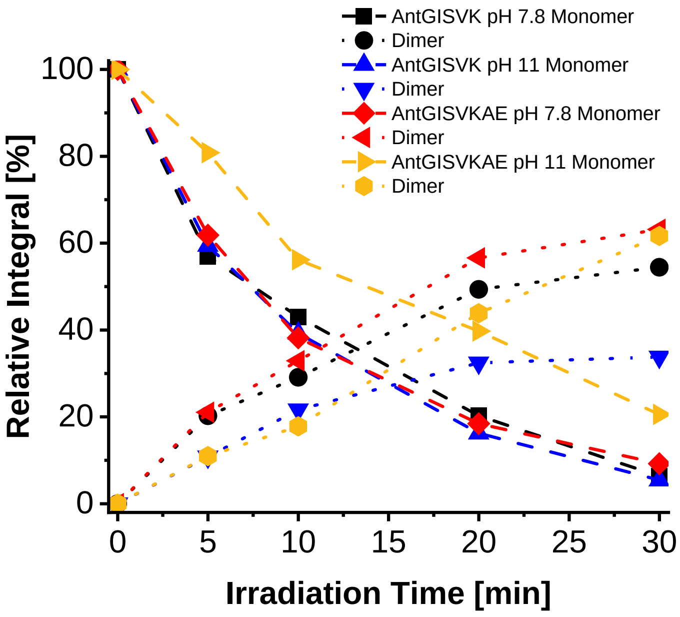

Figure 13: Relative integral of the anthracene monomer and dimer signals of **1** and **2** in 80% DMSO-d_6 and 20% NH_4HCO_3 buffer in H_2O (10 mM, pH 7.8 or pH 11) by ^1H NMR (700 MHz, 298 K).

3.2.1 HMBC spectroscopy

3.7 mg of peptide **2** were dissolved in 0.8 mL DMSO-d_6 and irradiated at 365 nm for 30 min. Then $^1H,^{13}C$-HMBC spectroscopy was measured.

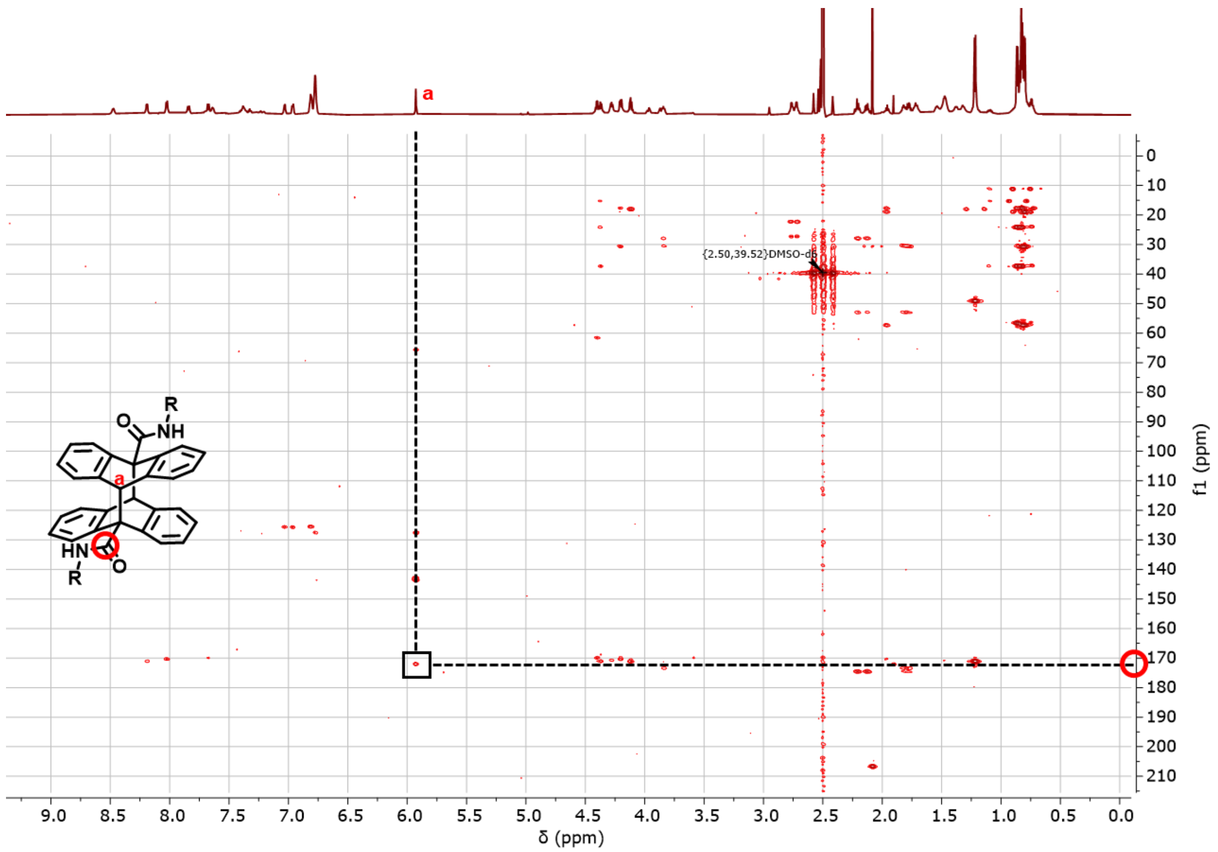

Figure 14: HMBC ^1H-^{13}C-NMR of **1** in DMSO-d$_6$ after 30 min irradiation at 365 nm showing a correlation between the bridge proton at 5.8 ppm and the 13C-signal at 172 ppm corresponding to the carbonyl carbon of the amide.

3.2.2 Analysis of side reaction

2.1 mg of 9-anthracene carboxylic acid were dissolved in 600 µL DMSO-d$_6$/D$_2$O-Phosphate buffer (10 mM, pH 7.4) 8/2 and irradiated for 30 min at 365 nm. Then ^1H-NMR was measured. Besides remaining monomer and the photodimer, there is 9,10-anthraquinone generated as a side product. The signals match with literature.[1]

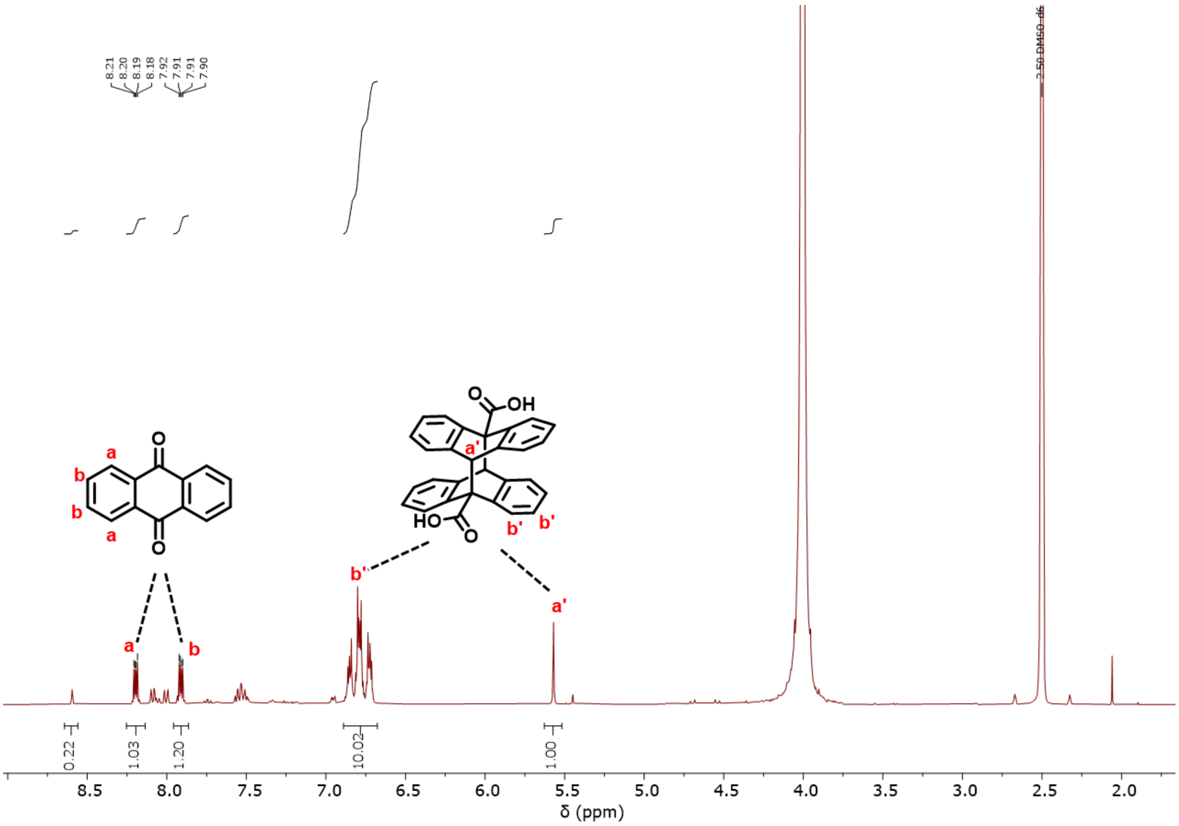

Figure 15: : ^{1}H-NMR of 9-anthracene carboxylic acid after 30 min irradiation at 365 nm, showing the formation of dimer and anthraquinone as side product (δ 8.20 (dd, J = 5.8, 3.4 Hz, 1H), 7.91 (dd, J = 5.8, 3.3 Hz, 1H)).

Additionally 2.1 mg 9-anthracene carboxylic acid were dissolved in the following solvents, irradiated for 5 min at 365 nm and then ^{1}H-NMR was measured:

80% THF-d_8, 20% D_2O-Phosphate buffer (10 mM, pH 7.4)

80% DMSO-d_6, 20% D_2O-Phosphate buffer (10 mM, pH 7.4)

50% DMSO-d_6, 50% $CDCl_3$

Ratios of product, starting material and side product were then calculated by integration of the respective peaks.

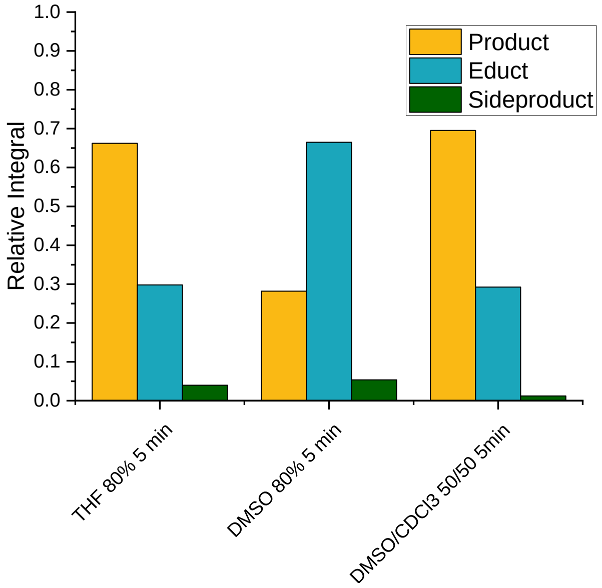

Figure 16: Relative integrals of the 5 min irradiation of 9-anthracene carboxylic acid in different solvent compositions according to ^{1}H-NMR measurements.

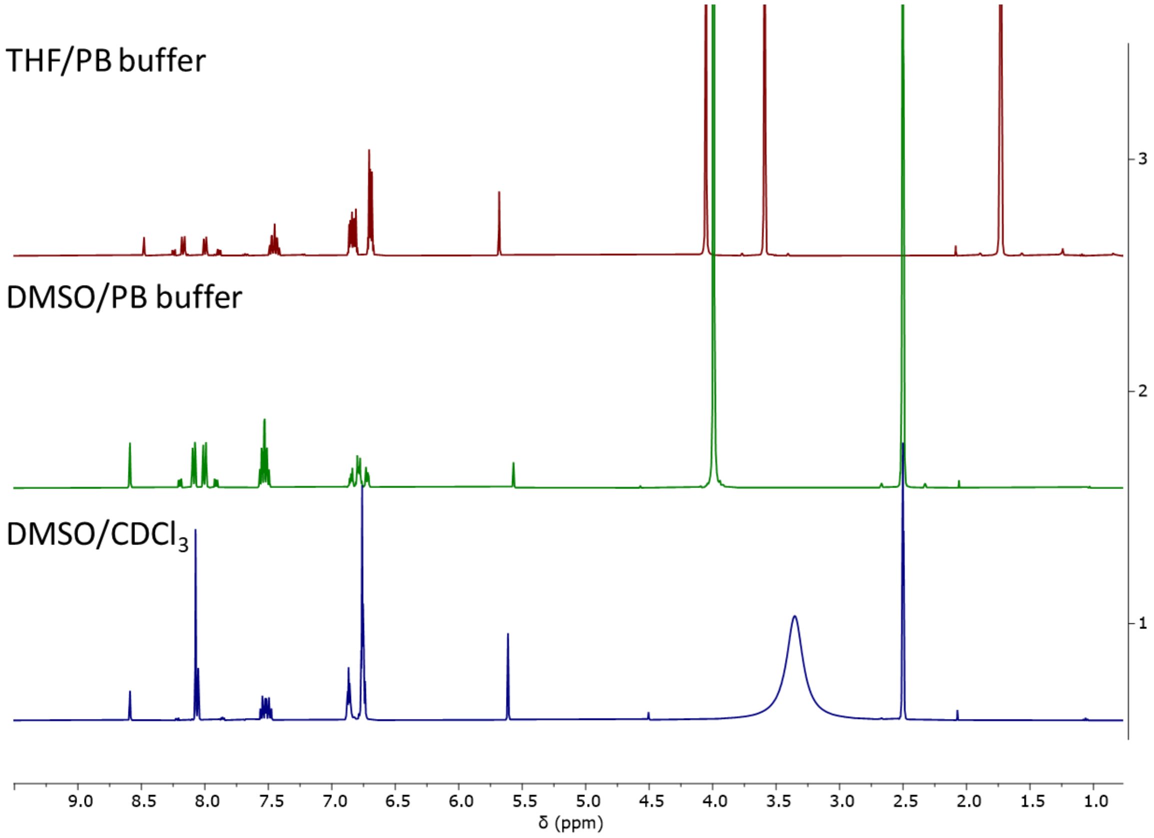

Figure 17: ^{1}H-NMR of 9-anthracene carboxylic acid after 5 min of irradiation at 365 nm in different solvents.

3.3 Transmission electron microscopy

3.3.1 Non-irradiated samples

1 (100 µM) was dissolved in 5% DMSO and 95% NH_4HCO_3 buffer (10 mM, pH 7.8) and incubated overnight at 25°C while shaking at 300 rpm. Then TEM grids were prepared by pipetting 3 µL solution onto a Formvar-coated copper grid and incubated for 5 min. After the incubation, the solutions were removed with filter paper, and the grids were stained with 7 µL 4% uranyl acetate solution for 2.5 min. The grids were washed three times with MilliQ water and dried before being measured. The procedure was conducted for **1** in 5% DMSO and 95% NH_4HCO_3 buffer (10 mM, pH 11) and for **2** at both pH values.

3.3.2 Irradiated samples

1 (100 µM) was dissolved in 5% DMSO and 95% NH_4HCO_3 buffer (10 mM, pH 7.8) and incubated overnight at 25°C while shaking at 300 rpm. The solution was then transferred to a glass vial equipped with a stirring bar and covered with a blanket of argon. It was then irradiated at 365 nm for 10 min while stirring at 300 rpm and again incubated overnight at 25°C. Then TEM grids were prepared by pipetting 3 µL solution onto a Formvar-coated copper grid and incubated for 5 min. After the incubation, the solutions were removed with filter paper, and the grids were stained with 7 µL 4% uranyl acetate solution for 2.5 min. The grids were washed three times with MilliQ water and dried before being measured. The procedure was conducted for **1** in 5% DMSO and 95% NH_4HCO_3 buffer (10 mM, pH 11) and for **2** at both pH values.

3.3.3 Assembly in DMSO/$CHCl_3$

2 (100 µM) was dissolved in 1% DMSO and 99% $CHCl_3$ and incubated overnight. Then TEM grids were prepared by pipetting 3 µL solution onto a Formvar-coated copper grid in a chloroform saturated atmosphere and incubated for 1.5 min. After the incubation, the solutions were removed with filter paper, and the grids were stained with 7 µL 4% uranyl acetate solution for 2.5 min. The grids were washed three times with MilliQ water and dried before being measured.
Then, **2** (100 µM) was dissolved in DMSO (10 mM) and irradiated for 10 min at 365 nm. The solution was then added to Chloroform to yield a 100 µM peptide solution in 1% DMSO and 99% $CHCl_3$. The sample was then incubated and measured as stated above.

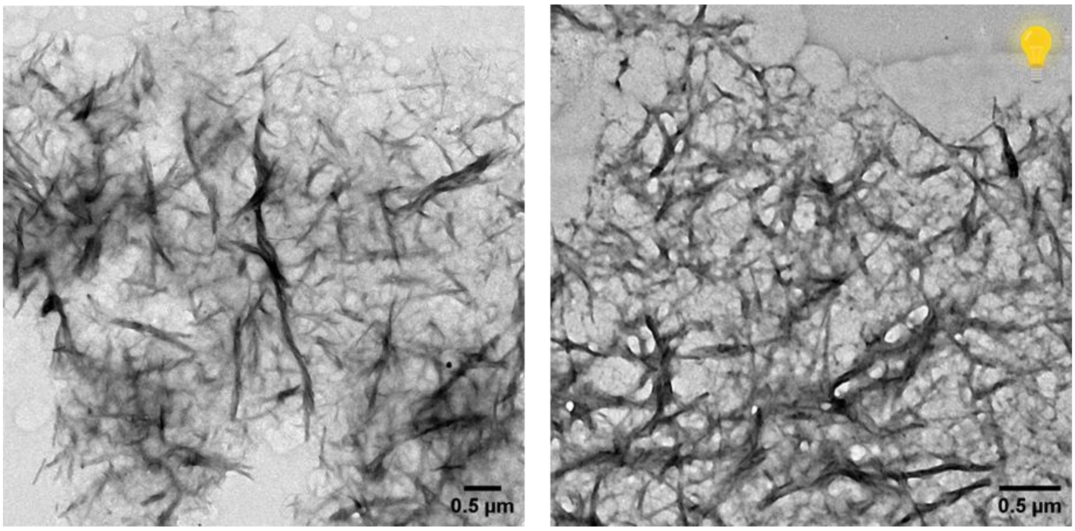

Figure 18: TEM image of 2 (100 µM) in 1/99 DMSO/CHCl3 before (left) and after 10 min of irradiation at 365 nm (right). All TEM scale bars are 0.5 µm.

3.4 Circular Dichroism

1 (100 µM) was dissolved in 5% DMSO and 95% NH_4HCO_3 buffer (10 mM, pH 7.8) and incubated overnight at 25°C while shaking at 300 rpm. CD spectra were recorded at wavelength from 300 to 185 nm with a bandwidth of 1 nm, data pitch of 0.2 nm and a scanning speed at 20 nm/min from 20°C. Spectra were measured three times. The procedure was conducted for **1** in 5% DMSO and 95% NH_4HCO_3 buffer (10 mM, pH 11) and for **2** at both pH values.

Samples for irradiation were transferred to a glass vial equipped with a stirring bar and covered with a blanket of argon. They were each irradiated at 365 nm for 10 min while stirring at 300 rpm and again incubated overnight at 25°C. The samples were measured as described above.

3.5 Fourier-transform Infrared spectroscopy

1 (500 µM) or **2** (500 µM) was dissolved in 5% DMSO and 95% NH_4HCO_3 buffer (10 mM, pH 7.8) and incubated overnight at 25°C while shaking at 300 rpm. Each sample was then measured in a cell between two CaF_2 windows separated by a 100 µm Teflon spacer.

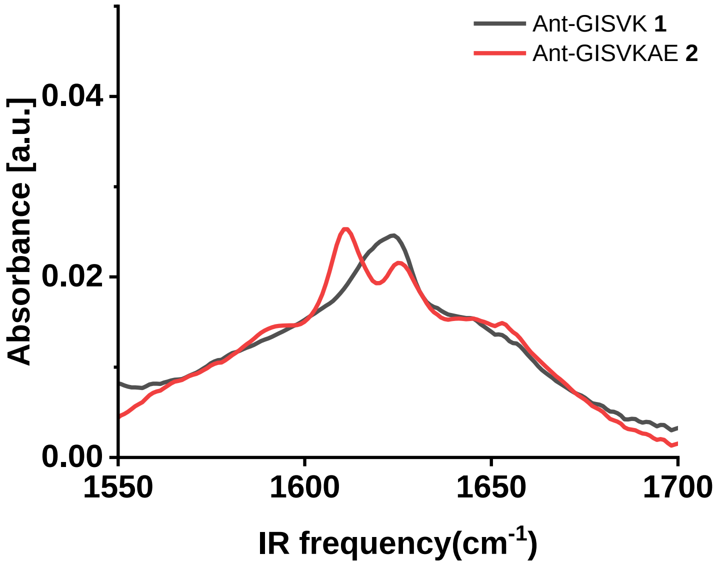

Figure 19: FTIR of $\mathbf{1_{pH7.8}}$ and $\mathbf{2_{pH7.8}}$ in 5% DMSO and 95% NH_4HCO_3 buffer (10 mM)

[1] M. Klaper, P. Wessig, T. Linker, *Chem. Commun.* **2016**, *52*, 1210–1213.
